# Biocontrol potential of a rhizosphere-associated *Bacillus amyloliquefaciens* F2 isolate against *Fusarium oxysporum* f. sp. *cubense* Tropical Race 4 in Pakistan

**DOI:** 10.3389/fmicb.2026.1957002

**Published:** 2026-10-20

**Authors:** Amna Shabbir, Sumaira Farrakh, Sobia Rubab, Somia Mehreen, Sehrish Shabbir, Sakina Benabdelkader, Nadeem Hassan, Tayyaba Yasmin, Mustafa Nawaz Shafqat, Huixiang Yin, Naitong Yu, Muhammad Nadeem Hassan

**Affiliations:** 1 Department of Biosciences, COMSATS University Islamabad, Islamabad, Pakistan; 2 Institute of Tropical Bioscience and Biotechnology, Chinese Academy of Tropical Agricultural Sciences, Haikou, China

**Keywords:** *Bacillus amyloliquefaciens*, biocontrol related genes, fusarium wilt, hydrolytic enzymes, promoting plant growth, volatile compounds

## Abstract

Fusarium wilt caused by *Fusarium oxysporum f.* sp. *cubense* is a serious fungal, soil-borne disease. It is also known as PANAMA disease, considered a major threat to the yield and quality of Banana worldwide. In this study, endophytic bacteria were isolated from soil, roots, and leaves of healthy banana plants growing in *Fusarium oxysporum* f. sp. *cubense* (Foc) tropical race 4 (TR4) infested soil. These endophytes were evaluated for their ability to inhibit the growth of Foc TR4. The results indicated that 7 out of 138 endophytic bacterial isolates showed significant inhibitory activity against mycelial development of *F. oxysporum* f. sp. *cubense*, with an inhibition rate of more than 50%. Isolate F2, which showed maximum inhibition (74%), showed 99.49% sequence identity with *Bacillus amyloliquefaciens strain seq. S1* based on 16S rRNA sequence. Biological and PCR assays demonstrated that the isolate F2 promotes plant growth by actively producing indole-3-acetic acid, siderophore, ammonia, and hydrolytic enzymes, including protease, pectinase, amylase, catalase, cellulase, and chitinase. It expresses biocontrol-related genes, including surfactin, fengycin, iturin, bacillomycin, bacillibactin, bacillaene, subtilosin, macrolactin, *yngG*, *yndJ*, biotin, difficidin, and bacilysin. The genes surfactin, fengycin, iturin, and bacillibactin in this bacterial isolate are upregulated when it interacts with Foc TR4. It also produces various volatile compounds that may inhibit the growth of Foc TR4. These findings demonstrate that the F2 isolate could be a potential candidate for controlling *Fusarium oxysporum* f. sp. *cubense* in banana.

## 1 Introduction

Bananas (*Musa* spp.) are among the most essential food and economic crops globally. With over 1,200 varieties, bananas are cultivated in more than 135 countries worldwide (Crane et al., 2006; Maseko et al., 2024). However, the Cavendish banana variety is preferred globally due to its high productivity and economic advantages (Yadav et al., 2021). This widespread monoculture, however, makes the crop highly susceptible to serious diseases, the most significant of which is Fusarium wilt (Dita et al., 2018; Staver et al., 2020).

Fusarium wilt in bananas is caused by the soil-borne pathogen *Fusarium oxysporum* f. sp. *cubense* (Foc) (Ploetz, 2006). This pathogen is classified into different physiological races based on its virulence to different banana cultivars in the field: Race 1 (R1) infects the Gros Michel (AAA), Silk, and Pome (AAB); Race 2 (R2) affects the Bluggoe (ABB); and Race 4 (R4) infects Cavendish cultivars (AAA group), along with all cultivars susceptible to Race 1 and Race 2 (Ploetz, 2015; Pegg et al., 2019). Race 4 is further divided into subtropical race 4 (STR4) and the highly aggressive tropical race (TR4) (Pegg et al., 2019). The Cavendish-driven export and its widespread cultivation have led to the rapid proliferation of *Fusarium oxysporum* f. sp. *cubense* TR4, resulting in the devastation of plantations within a short period and causing significant economic losses to landholders (Dita et al., 2018; Damodaran et al., 2020).

Since its emergence in 1967 in Taiwan, TR4 has spread across Southeast Asia, affecting countries such as China, India, Indonesia, Malaysia, the Philippines, Laos, Myanmar, Vietnam, and Pakistan. The pathogen has also spread intercontinentally, with reports of its presence in Australia, Jordan, Lebanon, Oman, Mozambique, Colombia, and Turkey (Ploetz, 2015; Ordoñez et al., 2016; Chittarath et al., 2018; Dita et al., 2018; Zheng et al., 2018; Damodaran et al., 2019). Fusarium wilt is considered a major disease of banana, causing annual losses of millions of US dollars and predicted to reach about US$10 billion globally by 2040 (Staver et al., 2020). In Pakistan, it was first found in 2012 in Thatta district of Hyderabad and reported in 2015 (Syed et al., 2015). The average disease incidence was reported as 17%, ranging from 2 to 42% in different regions (Koondhar et al., 2024).

Banana is a major fruit crop of Pakistan. It grew on about 40,106 hectares, with production of 311,166 tons in Neupane, 2025, mainly in Sindh province, where the soil and climatic conditions are favorable for its production (FAOSTAT Neupane, 2025). The major variety grown is Dwarf Cavendish (Basrai), which covers 90% of the area under cultivation, while a few more varieties have also been introduced recently. Most of the banana harvest is consumed locally, and the rest is exported to neighboring countries. Production on one ha can bring around Rs. 1.4 million (equivalent to $ 5,000), the highest among fruits grown in the country. Pakistan exported about 82,000 metric tons out of 311,166 metric tons of total production from 40,106 ha in Neupane, 2025 (FAO, 2026). In Sindh, all the varieties grown are Cavendish Banana, which is susceptible to Foc TR4. The emergence of Foc TR4 in Sindh is an alarming threat to the future of the banana industry in Pakistan (PARC report).

*Fusarium oxysporum* f. sp. *cubense* is particularly difficult to control as it is a soil-borne fungus with a long survival rate in the soil, lasting over 20 years, even in the absence of plant hosts (Pegg et al., 2019). Chemical control using fungicides has proven minimally effective (Jing et al., 2020). As a vascular pathogen, Foc escapes the reach of non-systemic fungicides, and the intensive use of these chemicals leads to pathogen resistance and increased environmental contamination (Neupane, 2025). Consequently, many researchers have shifted their focus to alternative methods such as selecting resistant varieties, crop rotation, and developing novel plant protectants to control Foc TR4 (Nel et al., 2007; Akila et al., 2011). However, breeding resistant varieties faces challenges due to the need for resistant germplasm screening, market acceptance of new varieties, the parthenocarpic nature of the plant, sterility issues, and poorly maintained seed sets (Zorrilla-Fontanesi et al., 2020).

Biological control is an eco-friendly strategy to manage soil-borne phytopathogens. Beneficial microorganisms are an important source of agricultural biocontrol agents. Recently, endophytes have received considerable attention for their potential to control fungal phytopathogens from inside the host plant (De Silva et al., 2019). When compared to artificial fertilizers and pesticides, the utilization of these microorganisms in managing plant diseases is more beneficial. *Bacillus* (Fan et al., 2023), *Trichoderma* (Damodaran et al., 2020), *Actinobacteria* (Wang et al., 2022), and *Streptomyces* (Chen et al., 2026) species have been screened for biological control of plant diseases due to their reduced cost.

Endophytic microbes directly resist the pathogen by competing with it for nutrients and sites and by the secretion of secondary metabolites. They indirectly show resistance to pathogens by inducing systemic resistance in plants, by enhancing the beneficial microbiota, by preventing the colonization of pathogens, and by improving plant growth (Lin et al., 2009). Using beneficial microbes is considered the most suitable method of controlling Foc TR4. These beneficial microbes can be used for disease suppression in infected soils.

This study aims to evaluate the biocontrol potential of bacteria associated with healthy banana plants growing in Foc TR4-infected fields and characterize the antagonistic and plant growth-promoting mechanisms for the control of Foc TR4.

## 2 Methodology

### 2.1 Sample collection

Eight samples of healthy banana plants were collected from infected fields of Tandojam Matiari Road Farm 1, Tandojam Matiari Road Farm 2, PARC Farm Nigab1 Variety (PFNVB) BB1, PARC Farm Nigab 1 Variety BSV (PFNSVB), Rasidi Farm, and Hanif Farm of Sindh Province (Figure 1).

**Figure 1:**
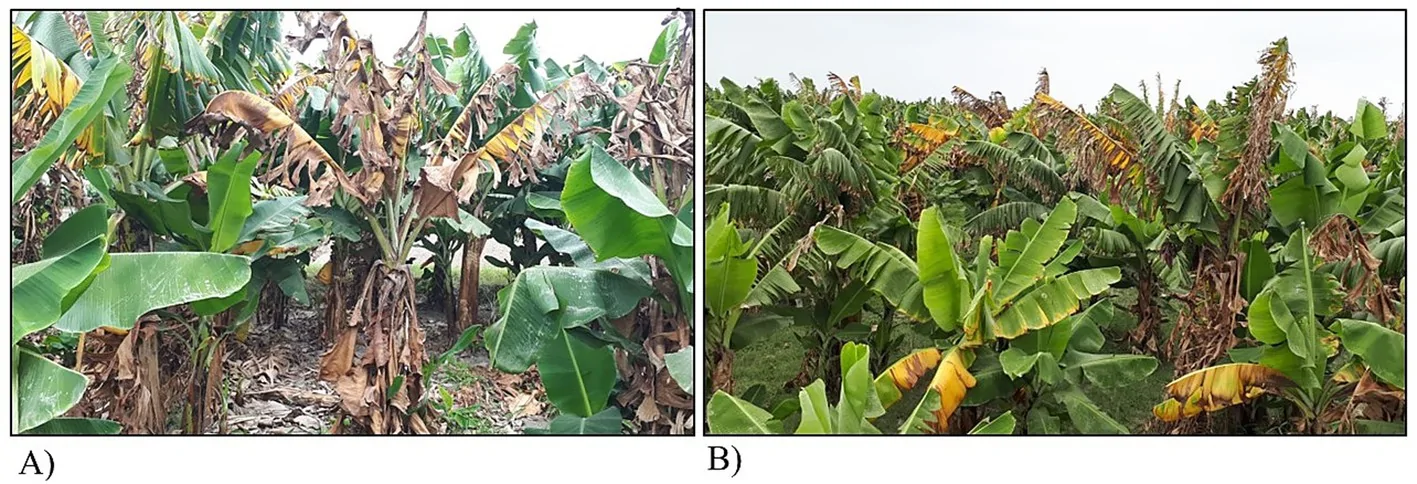
Field symptoms of Fusarium wilt in banana. **(A)** Foc TR4-infected banana plant showing typical disease external symptoms, wilting of leaves. **(B)** Banana farm infected by Foc TR4 showing infected banana plants surrounded by some healthy banana plants.

### 2.2 Isolation of rhizobacteria

Stock solutions of the rhizospheric soil were prepared by mixing 1 g of rhizospheric soil in 10 mL sterile phosphate-buffered saline (PBS). The serial dilution of stocks was performed up to 10^−6^. 100 μL of all the stock solutions and their dilutions were spread separately on Tryptone soya agar (TSA) plates by the spread plate technique and incubated at 27 °C for 24–48 h (Apastambh et al., 2016).

### 2.3 Isolation from root, leaves, and stem tissues

Plant tissues were surface sterilized completely by first washing them in tap water to remove all the dirt and soil, then they were dipped in 70% ethanol for 1 min, followed by washing with sterile water, then dipped again in 2.5% sodium hypochlorite for 1 min, washing with sterile water, and then dipped again in 70% ethanol for 30 s and washed again with sterile water carefully to remove all the ethanol and sodium hypochlorite traces. The tissues were air-dried and then ground well in phosphate-buffered saline (PBS) to make a homogeneous mixture. The serial dilution was performed, and all the stocks and their dilutions were spread on TSA plates by the spread plate technique and incubated at 30 °C for 24–48 h (Kumar et al., 2016; Singh et al., 2022).

### 2.4 Revival of FOC isolates

All 21 FOC TR4 isolates already available in the lab and confirmed by molecular identification were revived from soil stocks and purified on potato dextrose agar amended with 50 μg/mL kanamycin.

### 2.5 *In vitro* biocontrol potential of Bacteria

Antagonistic effects of bacterial isolates were checked by the dual culture method. In the dual culture method, a 0.9 mm fungal disc was placed in the middle of the Potato Dextrose Agar (PDA) plate, and bacteria were streaked on three sides of the Petri plate, leaving one side of the plate as a control. Co-inoculated plates were incubated at 28 °C for 7 days. Mycelial growth from four sides was measured on day 7. Percentage inhibition was calculated using the formula mentioned below (Hussein et al., 2024).


$$\%age\;inhibition=\left(\left(\frac{C-I}{C}\right)*100\right)$$

C = Average control mycelial length.

I = Average inhibited mycelial length.

### 2.6 Molecular identification

#### 2.6.1 DNA extraction

Following the antagonistic screening, the Fs bacterial isolate was selected for molecular identification. Bacterial broth of the F2 bacterial culture was used to extract DNA. The culture was centrifuged at 9000 g for 30 s, and the supernatant was discarded. The pellet was dissolved in 0.1X TE buffer, mixed with lysozyme (10 mg/mL), and incubated at 37 °C for 30 min. After incubation, Proteinase K (20 mg/mL) was added, and the solution was incubated again at 65 °C for 3 h. After incubation, 10% SDS was added and incubated again at 70 °C for 30 min. After that, 4% CTAB was added and incubated at 65 °C for 20 min. DNA was extracted by adding chloroform, vortexing thoroughly, and centrifuging at 13,000 rpm for 15 min. The supernatant was then transferred to a new tube, and an equal volume of isopropanol was added. The tube was incubated at −20 °C for 20 min and centrifuged at 16,000 g for 10 min. The pellet was washed with 70% ethanol and air-dried. Once the pellet was dried, it was dissolved in TE/RNAse (10 mg/mL RNase in 0.1X TE buffer) and stored at −20 °C for further use (Zeeshan Hyder et al., 2007).

#### 2.6.2 16S rRNA sequencing

The 16S rRNA gene was amplified by PCR with the help of universal primers for 16S rRNA: 27F (5′-GAGTTTGATCACTGGCTCAG-3′) and 1492R (5′-TACGGCTACCTTGTTACGACTT-3′). 50 ul reaction was prepared for each DNA sample by adding 10X PCR buffer (NH_4_SO_4_) (Thermo Scientific Lot 2884079), 10XdNTPs (Thermo Scientific), 25 mM MgCl_2_ (Thermo Scientific Lot 2887332), Taq Polymerase (Thermo Scientific Lot 2828197), PCR water, 20 pM Primer mix, and DNA. Aliquots of PCR reaction products were electrophoresed in 0.8% agarose. After confirmation, PCR products were purified and sent for sequencing to Macrogen Inc. (Korea). DNA Dragon version 1.9.2^1^ was used to assemble consensus sequences of the 16S rRNA gene of all bacterial isolates. The sequence of each isolate was compared with the sequences available in the NCBI database using the BLAST tool. The sequence was aligned and clustered using MEGA version 11.0.13.

### 2.7 Plant growth-promoting (PGP) traits analysis

#### 2.7.1 Production of IAA

To identify the indole acetic acid production ability of the F2 bacterial isolate, it was streaked on TSA medium to obtain a single colony and then incubated at 30 °C for 24 h. The next day, the single colony was then picked and inoculated into nutrient broth supplemented with L-tryptophan (200 μg/mL). It was incubated for 24 h at 30 °C with shaking at 150 rpm. The next day, the broth culture was centrifuged at 16,000 g for 10 min. The 2 mL of the whole supernatant was transferred into another tube and mixed with two drops of orthophosphoric acid and 4 mL of Salkowski reagent, which is composed of 50 mL of 35% perchloric acid and 1 mL of 0.5 M FeCl_3_ solution. The change in color will indicate the production of indole acetic acid (Singh et al., 2022).

#### 2.7.2 Phosphate Solubilization

Phosphate solubilization activity of F2 bacteria is identified by Pikovskaia’s agar by the dissolution of precipitated calcium phosphate (Ca_3_ (PO_4_)_2_). The bacterial isolate was aseptically inoculated in the center of the Pikovskaia’s agar plates containing 0.5% calcium phosphate (Ca_3_ (PO_4_)_2_) and incubated at 30 °C for 3–5 days. The development of a clear halo zone around the inoculated bacterial isolate confirmed phosphate solubilization activity (Sahoo, 2018).

#### 2.7.3 Siderophore production

The qualitative assay for siderophore production was carried out using the Chrome azurol sulfonate (CAS) agar plate method. CAS agar medium was prepared by dissolving 88 mL Basal agar with 2 mL of 50% glucose solution and 10 mL of CAS solution in a water bath set at 50 °C.

CAS indicator solution was prepared by dissolving three solutions. Solution I: 60.5 mg CAS in 50 mL distilled water; Solution II: 10 mL Fe (III) solution (27 mg FeCl_3_.6H_2_O along with 83.3 μL conc. HCl dissolved in 100 mL distilled water); Solution III: 72.9 mg HDTMA dissolved in 40 mL distilled water.

Basal agar medium was composed of MOPS (3 g), NaCl (0.05 g), KH_2_PO_4_ (0.03 g), NH_3_Cl (0.01 g), L-asparaginase (0.05 g), and agar (1.2 g) in 88 mL distilled water at pH 6.8. The single colony of bacterial isolate F2 was inoculated on CAS agar plates and incubated for 5 days at 30 °C. The development of clear halo zones indicates siderophore production (Elgazzar, 2017).

#### 2.7.4 Hydrogen cyanide (HCN) production

Freshly grown F2 bacterial isolates were streaked onto nutrient agar medium plates amended with 4.4 g L^−1^ glycine. A single sheet of filter paper (size 90 mm) was soaked with 0.5% picric acid solution prepared in 2% sodium carbonate and placed in the lid of each plate. All Petri plates were sealed with Parafilm and incubated at 30 °C for 7 days. A positive result was checked by color change in the filter paper from yellow to an orange-brown tone. The rich yellow color of the filter paper remaining unaltered following the development of bacteria indicates a negative test. Results were compared to the control and categorized as negative (−) for no color change, moderately positive (++) for orange color, and highly positive (+++) for red-brown color (Prasad and Dagar, 2014).

#### 2.7.5 Potassium solubilization

To evaluate potassium solubilization activity, the F2 bacteria were inoculated on Aleksandrov agar plates (Glucose (5 g), MgSO_4._ 7H_2_O (0.5 g), FeCl_3_ (0.1 g), CaCO_3_ (2 g), potassium aluminosilicate (2 g), CaPO_4_ (2 g), Agar (12 g), and distilled water (1,000 mL)). The plates were incubated for 3–5 days at 30 °C. The development of a clear halo zone around the inoculated bacterial isolate confirmed potassium solubilization activity (Parmar and Sindhu, 2013).

#### 2.7.6 Zinc solubilization

To determine the zinc solubilization activity of the F2 bacterial isolate, it was aseptically inoculated on an agar plate (Glucose (10 g), (NH_4_)_2_SO_4_ (1 g), KCl (0.2 g), K_2_HPO_4_ (0.1 g), MgSO_4_ (0.2 g), ZnO/ZnCO_3_/Zn_3_(PO4)_2_ (0.1%), Agar (12 g), and distilled water (1,000 mL)). The plate was incubated at 30 °C for 3–5 days. The development of clear halo zone around the inoculated bacterial isolate confirmed zinc solubilization activity (Othman et al., 2022).

#### 2.7.7 Ammonia production

The single colony of the F2 bacterial isolate was inoculated into peptone water broth (peptone; 10 g, NaCl; 5 g, distilled water; 1,000 mL) and incubated at 30 °C for 24 h at 120 rpm. Supernatant (2 mL) was collected in separate test tubes, and 1 mL of Nessler’s reagent (KI; 70 g, HgCl_2_; 100 g, NaOH; 160 g, and distilled water; 1,000 mL) was added to detect the color change. The color change from yellow to reddish orange determines the ammonia production ability of bacteria (Singh et al., 2014).

### 2.8 Hydrolytic enzyme assay

The hydrolytic enzyme assay (amylase, protease, pectinase, chitinase, cellulase, lipase, and esterase enzymes) was performed on solid media. F2 bacterial isolate was grown on TSA to obtain a single colony for all enzyme assays. Each enzyme assay was performed in triplicate.

#### 2.8.1 Amylase assay

For amylase activity, the single colony of the F2 bacterial isolate was inoculated onto a nutrient agar plate supplemented with 0.2% soluble starch and incubated at 30 °C for 24 h. After incubation, the plates were flooded with Lugol’s iodine; the excess stain was washed away, and the formation of clear halos around the colony indicates positive activity (Singh et al., 2022).

#### 2.8.2 Protease assay

The proteolytic activity of the F2 bacterial isolate was analyzed on agar media supplemented with 1% skimmed milk powder. The bacteria were inoculated and then incubated at 30 °C for 48 h. The development of clear halos around the colony indicates positive proteolytic activity (Jalgaonwala and Mahajan, 2011; Singh et al., 2022).

#### 2.8.3 Pectinase assay

The pectinase activity of the F2 bacterial isolate was analyzed by inoculating the bacteria onto pectin agar medium (NaNO3; 0.1, KCl; 0.1, K2HPO4;0.1, MgSO4; 0.05, Yeast extract; 0.05, pectin; 1, agar; 1.2). The plates were incubated at 30 °C for 24 h. After incubation, the plates were flooded with Lugol’s iodine and incubated for 5 min in a dark place. After incubation, they were washed with distilled water and then analyzed for the formation of clear halos, which are indicative of pectinase activity (Jalgaonwala and Mahajan, 2011).

#### 2.8.4 Esterase assay

To check the esterase activity, F2 bacterial isolate was inoculated on media (Peptone; 1, Sodium chloride; 0.5, Calcium Chloride dihydrate; 0.01, agar; 1.2(g/100 mL)). The plates were incubated at 30 °C for 48–72 h. The precipitation around the colony is indicative of esterase activity (Jalgaonwala and Mahajan, 2011).

#### 2.8.5 Chitinase assay

The chitinase activity of the F2 bacterial isolate was analyzed by streaking it on chitin agar medium (Colloidal Chitin; 1, Peptone; 0.5, Yeast extract; 0.5, Potassium dihydrogen phosphate; 0.1, Magnesium sulfate; 0.01, agar; 1.2 (g/100 mL)). The colloidal chitin was prepared by dissolving 1 g chitin flakes in 20 mL of concentrated HCl with continuous stirring for about 4 h. When the thick, slurry-like mixture formed, it was transferred to 250 mL of chilled distilled water with continuous stirring. After 4 to 5 h of stirring, it was filtered using the LCCM (Lab Coat Clothing Material) method, in which LCCM was used as a physical filter element. The filtrate was dried completely and then weighed to prepare chitin agar medium. The bacteria were inoculated on chitin agar and incubated for 3 to 5 days at 30 °C. After incubation, Lugol’s iodine was poured on the colonies to identify the zone formed, which indicates the activity of chitinase (Mohandas et al., 2018).

#### 2.8.6 Cellulase assay

For cellulase activity, F2 bacterial isolate was inoculated onto the medium containing K_2_HPO_4_; 0.7 g, KH_2_PO_4_; 0.2 g, MgSO_4_; 0.01 g, yeast extract 0.1 g, sodium citrate; 5 g, carboxymethylcellulose (CMC) 1% and agar 1.2 g dissolved in 100 mL distilled water. The plates were incubated at 30 °C for 3–5 days. At the end of the incubation, Lugol’s iodine solution was poured onto Petri dishes and kept for incubation for 5 min. Then the Petri dishes were washed with distilled water to remove excess dye. Colonies with a halo zone around them were evaluated as cellulase-positive (Abu-Gharbia et al., 2018).

#### 2.8.7 Lipase assay

To evaluate lipolytic activity, the F2 bacterial isolate, purified bacterial colony, was inoculated on media containing Tryptone; 1.0 g, Yeast extract; 0.5 g, NaCl: 1 g, Olive oil; 1%, and agar; 1.2 g dissolved in 100 mL distilled water. Olive oil was added to the autoclaved medium through a 0.22 μm filter. The plate was incubated at 30 °C for 48–72 h. The formation of halo zones indicates lipase activity (Prasad and Dagar, 2014).

#### 2.8.8 Catalase assay

For the catalase assay, a drop of 5% Hydrogen peroxide was added to the glass slide. The single colony of F2 was mixed with the hydrogen peroxide on the glass slide. The bubble formation indicates catalase activity (Prasad and Dagar, 2014).

### 2.9 PCR confirmation of biocontrol-related genes

The extracted bacterial genomic DNA was screened for the presence of 13 biocontrol-associated genes, including *iturin*, *surfactin*, *bacillomycin*, *difficidin*, *macrolactin*, *fengycin*, *bacillibactin*, *biotin*, *bacilysin*, *yngG*, *yndJ*, *bacillaene*, and *subtilosin*. The DNA was amplified at two different annealing temperatures, i.e., 56 °C and 52 °C (Table 1). A 10 μL PCR reaction was prepared for each gene, comprising 7 μL master mix, 2 μL DNA, and 1 μL primer mix (as described in section 2.6.2).

**Table 1:** Primer sequences of biocontrol-related genes.

Sr. no	Metabolites	Genes	Primer sequence (5′–3′)	Tm	Product size (bp)	References
1.	Surfactin	*srfAA*	F: GAAAGAGCGGCTGCTGAAAC R: CCCAATATTGCCGCAATGAC	56 °C	273	Joshi and McSpadden Gardener (2006), Fan et al., (2021b), Li et al. (2021)
2.	Iturin	*ituC*	F: TTCACTTTTGATCTGGCGAT R: CGTCCGGTACATTTTCAC	52 °C	575	Joshi and McSpadden Gardener (2006), Fan et al. (2021b), Li et al. (2021)
3.	Fengycin	*fenD*	F: CCTGCAGAAGGAGAAGTGAAG R: TGCTCATCGTCTTCCGTTTC	56 °C	293	Joshi and McSpadden Gardener (2006), Fan et al. (2021b), Li et al. (2021)
4.	YngG	*yngG*	F: GAACTGTCCGAAACATGTCCG R: CTGAGCTCTTGAACGGTCCGG	52 °C	372	Joshi and McSpadden Gardener (2006), Fan et al. (2021b), Li et al. (2021)
5.	YndJ	*yndJ*	F: CAGAGCGACAGCAATCACAT R: TGAATTTCGGTCCGCTTATC	56 °C	212	Joshi and McSpadden Gardener (2006), Fan et al. (2021b), Li et al. (2021)
6.	Bacillomycin D	BamD	F: AGTCTAAGTATTGGCGAAACGA R: ATTATGCTGAAAGTGAAGGGCG	56 °C	448	Fan et al. (2021b), Li et al. (2021)
7.	Bacillibactin	*dhbA*	F: TCGGATTTCTTTTGCCACTTGG R: ATAACGGGCGCTCCTTCGGTTC	56 °C	440	Fan et al. (2021b), Li et al. (2021)
8.	Bacilysin	*bac*	F: TGAAGGGACAAGTAGTGAGTAC R: GATAGGAGACGGGTGGGATA	52 °C	317	Fan et al. (2021b), Li et al. (2021)
9.	Bacillaene	*bae*	F: TTGTGCGGTCGTGTATGAACAG R: TCGCCATCGAGATTAAGAAATT	56 °C	488	Fan et al. (2021b), Li et al. (2021)
10.	Biotin	*bioA*	F: TTCCACGGCCATTCCTATAC R: TTTGTCCCCTTATCCTGCAC	52 °C	210	Joshi and McSpadden Gardener (2006), Fan et al. (2021b), Li et al. (2021)
11.	Subtilosin	*sboA*	F: TCGGTTTGTAAACTTCAACTGC R: GTCCACTAGACAAGCGGCTC	56 °C	334	Fan et al. (2021b), Li et al. (2021)
12.	Difficidin	*dfn*	F: AGTAGTTTTTCTCATCGGTCTG R: GGCTCCTTATATTGGGGCATTC	52 °C	574	Fan et al. (2021b), Li et al. (2021)
13.	Macrolactin	*mln*	F: GCTGATGAACTGATAACAACCG R: CATTACGAAGCAAAATAAAAAA	56 °C	320	Fan et al. (2021b), Li et al. (2021)

#### 2.9.1 Expression analysis of biocontrol-related genes

RNA was extracted from F2 bacterial isolate grown in the presence and absence of Foc TR4, designated as control and treated samples, respectively. Following the treatment, the bacterial cells were harvested by centrifugation at 13,000 rpm. The resulting pellet of control and treated bacterial samples was dissolved in lysis buffer (0.1X TE, 10% SDS, 10mg/ml proteinase K and 10mg/ml lysozyme) and then incubated at 37⁰C temperature for 30 min. After incubation, TRIzol reagent (200 ul) was added to the mixture, and samples were incubated for 5 min at room temperature. Chloroform (0.2vol.) was then added and mixed thoroughly, and samples were incubated again for 15 min at room temperature. The samples were centrifuged at 12,000 rpm for 15 min at 4 °C. Then the upper layer was transferred into a new Eppendorf tube. The RNA was extracted by adding (0.5 vol.) isopropanol and incubated at -20 °C for 2 h. The samples were then centrifuged at 10,000 rpm for 10 min at 4 °C. The resulting pellet was washed with 70% ethanol. The pellet was air-dried and dissolved in nuclease-free water. In order to remove traces of genomic DNA, extracted RNA was treated with DNase I (2,500 U/μl) in DNase buffer (10 mM Tris–HCl, 2.5 mM MgCl_2_, and 0.5 mM CaCl_2_ with pH of 7.6). The reaction mixture was incubated at 37 °C for 15 min. DNase I activity was terminated by adding 0.5 M EDTA followed by incubation at 65 °C. The purified RNA pellet was dissolved in DEPC water and stored at -20 °C (Villa-Rodríguez et al., 2018).

Thermo Scientific Revert Aid RT Kit was used to synthesize cDNA from RNA. Briefly, 10 μL of RNA sample was mixed with 1 μL of Random Hexamer primers and 1 μL of nuclease-free water. Tubes were incubated at 65 °C for 5 min. Then 4 μL of 5X reaction buffer, 1 μL of RiboLock RNase Inhibitor (20 U/μL), 2 μL of 10 mM dNTP Mix, and 1 μL of RevertAid M-MuLV RT (200 U/μL) were added to make the final volume of 20 μL. RT-qPCR amplification was performed for 10 μL of volume of each reaction sample, with SybrGreen qPCR Master Mix (Takara, China). The reaction was prepared by adding 5 μL of SybrGreen qPCR Master Mix, 2 μL of nuclease-free water, 1 μL of gene-specific primer mix, and 2 μL of cDNA. The thermal cycling parameters were 5 min at 94 °C, 35 cycles of 15 s at 94 °C,1 min at 52 °C, and 30 s at 72 °C. (Zhao et al., 2007). Three technical replicates for each treatment were used. RNA polymerase subunit (*Rop*B) (F: 5’AGTATCCCGTTGAAGAGTCAAAAGA-3′) and (R: 5’CAAGCTGAGATACGATAACACGTTC-3′) were used as housekeeping genes to normalize the data. The mean cyclic threshold (Ct) values of technical replicates were used to measure the expression of the gene under specific treatment. The specificity of each reaction was also analyzed using the melt curve analysis. Relative abundance of the transcript was calculated using the Ct ^(2−ΔΔCt)^ (Livak and Schmittgen, 2001).

### 2.10 Aseptic filtrate analysis

#### 2.10.1 Preparation of aseptic filtrate

To prepare cell-free bacterial culture filtrate of selected bacteria, F2, the single colony was inoculated in LB medium, and it was incubated for 24 h at 30 °C and 180 rpm. After 24 h, 100 μL of overnight culture was transferred into 500 mL of fresh LB broth. The mixture was incubated once again for six days under the same conditions. After the incubation, the broth culture was centrifuged at 10,000 rpm for 15 min. The supernatant was transferred to another tube, and it was filtered through an aseptically sterilized bacterial filter with a 0.22 μm pore size (Sun et al., 2023). To confirm the absence of viable bacterial cells, an aliquot of the cell-free filtrate was plated on LB agar plates and incubated at 30 °C for 24–48 h.

#### 2.10.2 *In vitro* antifungal assay of aseptic filtrate

The antifungal activity of the aseptic filtrate was evaluated using the agar well diffusion method. PDA agar plates were prepared, and three wells of approximately 7.5 mm in diameter were aseptically punched at equal distances. Then,100 μL of cell-free filtrate was added to the wells. Once the cell-free filtrate was completely diffused into the agar, the mycelial plug of *Fusarium oxysporum* f. sp. *cubense* Tropical Race 4 (Foc TR4) was placed in the center of the plates. The plates were incubated at 30 °C for 7 days. Mycelial growth from four sides was measured on day 7. Percentage inhibition was calculated using the formula mentioned below.


$$\%age\;inhibition=\left(\left(\frac{C-I}{C}\right)*100\right)$$

C = Average control mycelial length.

I = Average inhibited mycelial length.

#### 2.10.3 Gas chromatography–mass spectrometry (GC–MS)

Secondary metabolite profiling of the selected bacterial isolate was performed using gas chromatography- mass spectrometry (GC–MS). Three groups were used for this profiling. The first group consisted of the fungus: Foc TR4; the second group consisted of only the selected bacteria (F2); and the third group consisted of the F2 + Foc TR4 interaction. The sample of the first group was prepared by inoculating the fungus (Foc TR4) in potato dextrose broth PDB and incubating at 30 °C for 7 days. To prepare the sample of the second group, bacteria (F2) were inoculated in LB broth and incubated at 30 °C with shaking at 120 rpm for 7 days. For the third group, the Foc TR4 was first inoculated in PDB and incubated at 30 °C for 24 h. After 24 h, the bacteria were inoculated into the same culture and incubated at 30 °C for 6 days. After the incubation, the samples from all groups were centrifuged at 12,000 rpm for 10 min at 4 °C, and supernatants were collected in separate flasks. An equal amount of acetone was added to the supernatants and thoroughly mixed. The mixture was incubated overnight at -20 °C. The samples were concentrated by evaporation in a water bath at 45 °C. The residue was then dissolved in 1 mL methanol (Naveed et al., 2023). The prepared samples of each group were submitted to an external analytical facility for GC–MS profiling. Metabolite profiling was performed using an Agilent Technology (USA) GC–MS system equipped with a 7890B gas chromatograph and a 5977B mass spectrometer. The obtained mass spectra were compared with reference spectra in the relevant mass spectral library to identify the detected compounds.

### 2.11 Statistical analysis

The triplicate data obtained from the dual culture assay were analyzed using analysis of variance (ANOVA) to determine the effect of biocontrol bacteria on pathogen growth. The mean values with standard deviations were calculated, and the significant treatments were identified via Duncan’s Multiple Range Test (DMRT) at the 5% significance level (*p* ≤ 0.05). The statistical analysis was performed using the software Jamovi and its Rj module.

## 3 Results

A total of 138 bacteria were isolated from all collected tissues and rhizospheric soil samples. A total of 82 morphologically distinct bacteria were collected from rhizospheric soil samples; 14 bacteria were isolated from root tissues, while 42 bacterial isolates were isolated from the stem samples. All these isolates were used for *in vitro* evaluation of their biocontrol potential against Foc TR4 (Supplementary Table 1).

### 3.1 *In vitro* biocontrol potential of bacteria

From the rhizosphere, 50% of the bacterial isolates inhibited Foc TR4. The inhibition percentage ranges from 10 to 74%. The 4 isolates A3 (52.20%), F2 (67.30), F8 (50.33%), and R18 (53.00%) showed maximum inhibition on average. From root tissue, all 14 isolates showed inhibition toward Foc TR4. The percentage inhibition ranged from 10 to 60%. A41b showed the maximum percentage inhibition of 60.04% on average. From the stem tissue, 45% of the isolates showed inhibition of Foc TR4. The inhibition percentage ranged from 10 to 60%. C1(56.59%) and C2 (58.46%) showed maximum inhibition on average among all isolates.

Duncan’s Multiple Range Test (DMRT) revealed the differences among the bacterial isolates. F2 showed strong activity against 19 out of 21 fungal isolates. A41b and C2 also showed strong antagonistic activity against 4 and 7 of the 21 fungal strains, respectively, while F8, R18, and A3 showed lower antagonistic activity (Figure 2 and Table 2).

**Figure 2:**
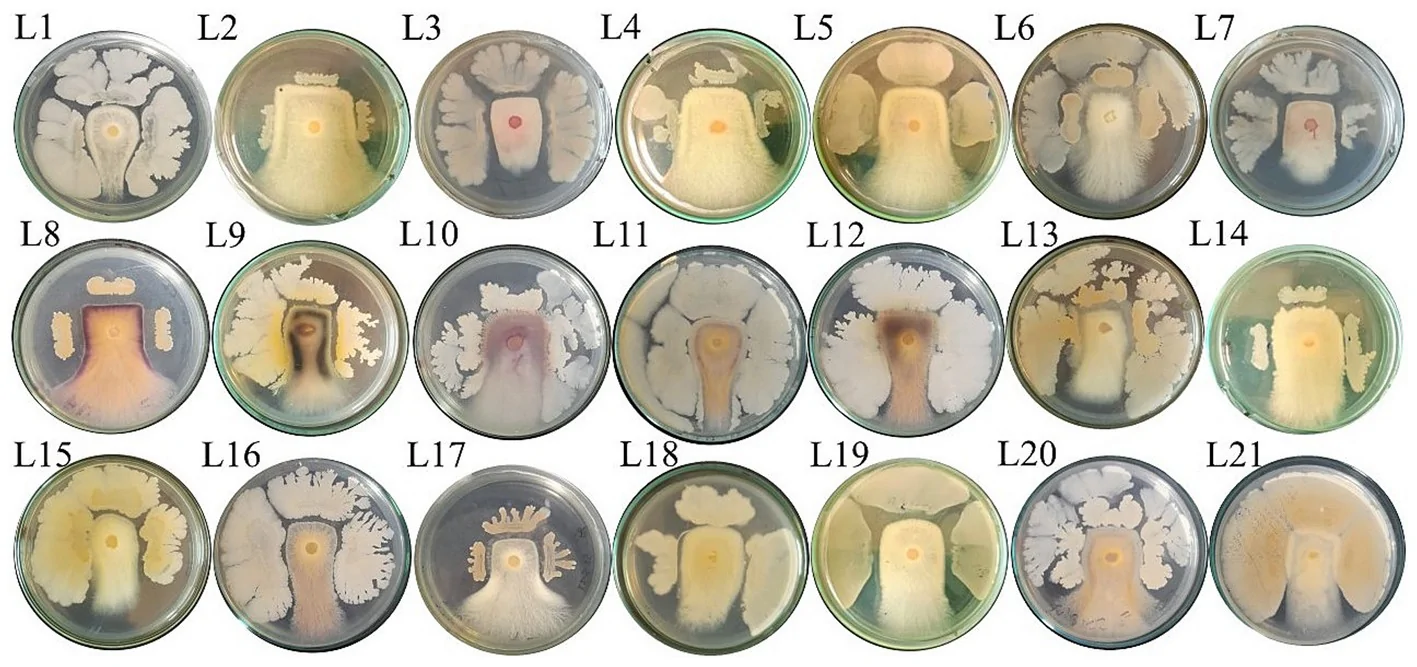
*In vitro* antagonistic activity of endophytic bacterial isolate F2 against 21 Foc TR4 isolates. It shows the interaction between isolate F2 and each fungal isolate with varying degrees of antifungal activity, ranging from moderate inhibition to high inhibition.

**Table 2:** Antagonistic activity of bacterial isolates against Foc TR4 isolates.

Sr. no	Fungal isolates	Bacterial isolates						
		Soil				Roots	Stem	
		A3	F2	F8	R18	A41b	C1	C2
1	P.TH4-L1-DAS	42.67 ± 2.31^c^	65.66 ± 1.59^a^	18.63 ± 7.40^d^	43.14 ± 4.49^cb^	50.62 ± 4.28^b^	47.78 ± 3.85^cb^	60.74 ± 1.28^a^
2	P.TH5-L2-DAS	51.76 ± 0.00^b^	63.57 ± 3.57^a^	44.05 ± 2.06^c^	44.44 ± 2.41^c^	63.33 ± 1.44^a^	51.85 ± 3.70^b^	43.48 ± 0.00^c^
3	P.TH6-L3-DAS	54.17 ± 1.44^d^	67.46 ± 1.37^a^	43.75 ± 3.12^e^	58.14 ± 2.33^c^	65.12 ± 0.00^ba^	58.73 ± 1.37^c^	62.09 ± 0.00^b^
4	P.TH7-L4-DAS	52.86 ± 2.86^b^	65.93 ± 3.39^a^	51.11 ± 2.22^b^	42.67 ± 2.31^c^	65.00 ± 0.00^a^	65.93 ± 1.28^a^	42.03 ± 5.02^c^
5	P.TH8-L5-DAS	52.86 ± 2.86^c^	65.00 ± 2.50^a^	56.25 ± 3.12^cb^	56.79 ± 2.14^cb^	58.33 ± 2.89^b^	52.78 ± 2.41^c^	46.67 ± 2.31^d^
6	P.TH9-L6-DAS	58.33 ± 1.44^b^	65.24 ± 1.65^a^	54.04 ± 1.52^c^	52.12 ± 1.31^c^	64.86 ± 2.70^a^	44.44 ± 1.92^d^	64.29 ± 2.38^a^
7	P.TH10-L7-MF	42.99 ± 3.92^c^	64.29 ± 2.86^a^	52.02 ± 2.69^b^	61.48 ± 1.28^a^	31.82 ± 0.00^d^	62.96 ± 4.63^a^	62.22 ± 2.22^a^
8	P.TH11-L8-MF	47.78 ± 6.94^d^	66.67 ± 2.22^a^	50.00 ± 2.50^d^	52.96 ± 1.28^dc^	60.00 ± 2.22^b^	52.56 ± 2.22^dc^	57.41 ± 1.28^cb^
9	P.TH12-L9-DAS	54.07 ± 1.28^e^	74.24 ± 1.31^a^	57.41 ± 1.28^ed^	56.67 ± 3.82^ed^	68.25 ± 2.75^b^	60.00 ± 0.00^d^	64.07 ± 1.28^c^
10	P.TH13-L10-103MN	54.44 ± 1.92^b^	64.59 ± 5.18^a^	57.14 ± 0.00^b^	54.17 ± 1.44^b^	48.89 ± 1.92^c^	57.50 ± 0.00^b^	66.67 ± 1.44^a^
11	P.TH14-L11-103MN	48.89 ± 2.22^e^	72.60 ± 2.24^a^	54.44 ± 2.22^d^	55.19 ± 1.28^d^	64.17 ± 1.44^b^	60.49 ± 1.29^c^	61.85 ± 2.57^cb^
12	P.TH15-L12-103MN	52.70 ± 2.75^c^	61.52 ± 5.25^ab^	55.19 ± 1.28^c^	58.33 ± 1.44^cb^	54.44 ± 1.92^c^	58.33 ± 4.77^cab^	64.07 ± 1.28^a^
13	P.TH16-L13-103MN	54.44 ± 2.22^b^	68.53 ± 0.00^a^	50.00 ± 6.96^bc^	44.76 ± 3.30^c^	65.93 ± 1.28^a^	56.19 ± 4.36^b^	63.33 ± 1.44^a^
14	P.TH17-L14-103MN	58.10 ± 8.73^ba^	63.73 ± 1.70^a^	45.24 ± 0.01^c^	55.81 ± 0.00^b^	48.00 ± 0.01^c^	57.50 ± 0.00^ba^	60.00 ± 0.00^ba^
15	P.TH18-L15-103MN	51.26 ± 3.12^c^	69.17 ± 1.44^a^	57.46 ± 2.75^b^	56.67 ± 1.44^b^	56.67 ± 1.44^b^	60.00 ± 0.00^b^	59.17 ± 1.44^b^
16	P.TH19-L16-BPD	55.93 ± 1.28^c^	72.59 ± 1.28^a^	56.67 ± 2.22^c^	57.41 ± 1.28^c^	65.93 ± 5.13^b^	64.81 ± 1.28^b^	59.63 ± 3.39^c^
17	P.TH20-L17-BPD	58.89 ± 2.22^b^	71.85 ± 1.28^a^	48.00 ± 4.00^c^	51.48 ± 1.28^c^	70.37 ± 1.28^a^	64.04 ± 1.52^b^	59.52 ± 6.30^b^
18	P.TJ1-L18-KF	51.90 ± 0.00^b^	63.54 ± 6.51^a^	51.11 ± 1.92^b^	54.04 ± 1.52^b^	65.83 ± 1.44^a^	42.11 ± 5.26^c^	62.96 ± 3.21^a^
19	P.TJ2-L19-KF	55.56 ± 0.00^d^	64.76 ± 0.00^a^	43.18 ± 0.00^f^	46.51 ± 0.00^e^	55.56 ± 0.00^d^	61.36 ± 0.00^b^	59.09 ± 0.00^c^
20	P.TJ3-L20-KF	47.29 ± 1.80^d^	68.33 ± 1.44^a^	53.33 ± 1.44^c^	55.19 ± 1.28^c^	68.15 ± 2.57^a^	49.33 ± 2.31^d^	61.11 ± 2.22^b^
21	P.NS1-L21-SK	49.37 ± 1.28^e^	74.33 ± 0.00^a^	58.15 ± 1.28^dc^	55.21 ± 3.61^d^	69.63 ± 2.57^b^	60.37 ± 1.28^c^	47.44 ± 2.22^e^

This table shows the Mean ± Standard Deviation values with Duncan’s Multiple Range Test (DMRT) grouping. The same letters indicate no significant difference (*p* > 0.05) between isolates, whereas different letters show significant differences among the isolates.

### 3.2 Morphological and molecular identification of isolate F2

Following the antagonistic screening of the bacterial isolates, the F2 bacterial isolate was selected for the study. The single colonies of F2 after 24 h of incubation appeared off-white, rhizoid-shaped, and had a rough surface with undulate margins. The colonies were raised and dry, with a sticky texture (Table 3; Figure 3). The bacterial cells were Gram-positive and cocci-shaped, forming long chains. The BLAST analysis of the 16S rRNA gene sequence of F2 exhibited over 99.49% similarity with *Bacillus amyloliquefaciens* strain seq. S1. Phylogenetic analysis conducted with MEGA version 11.0.13 indicated that the isolate F2 was clustered within the *Bacillus amyloliquefaciens* clade and exhibited close genetic affiliation with the *Bacillus amyloliquefaciens* strain NBRC 15535, with a strong bootstrap value of 97% (Figure 4). The 16S rRNA gene sequence of the bacterial isolate F2 was submitted to the NCBI database, and the assigned accession number is OQ221535.

**Table 3:** Morphological characteristics of the strain F2.

Bacterial isolates	Morphological characterization					
	Margin	Color	Elevation	Texture	Shape	G-staining
F2	Undulate	Off white	Flat	Sticky	Rhizoid	+

**Figure 3:**
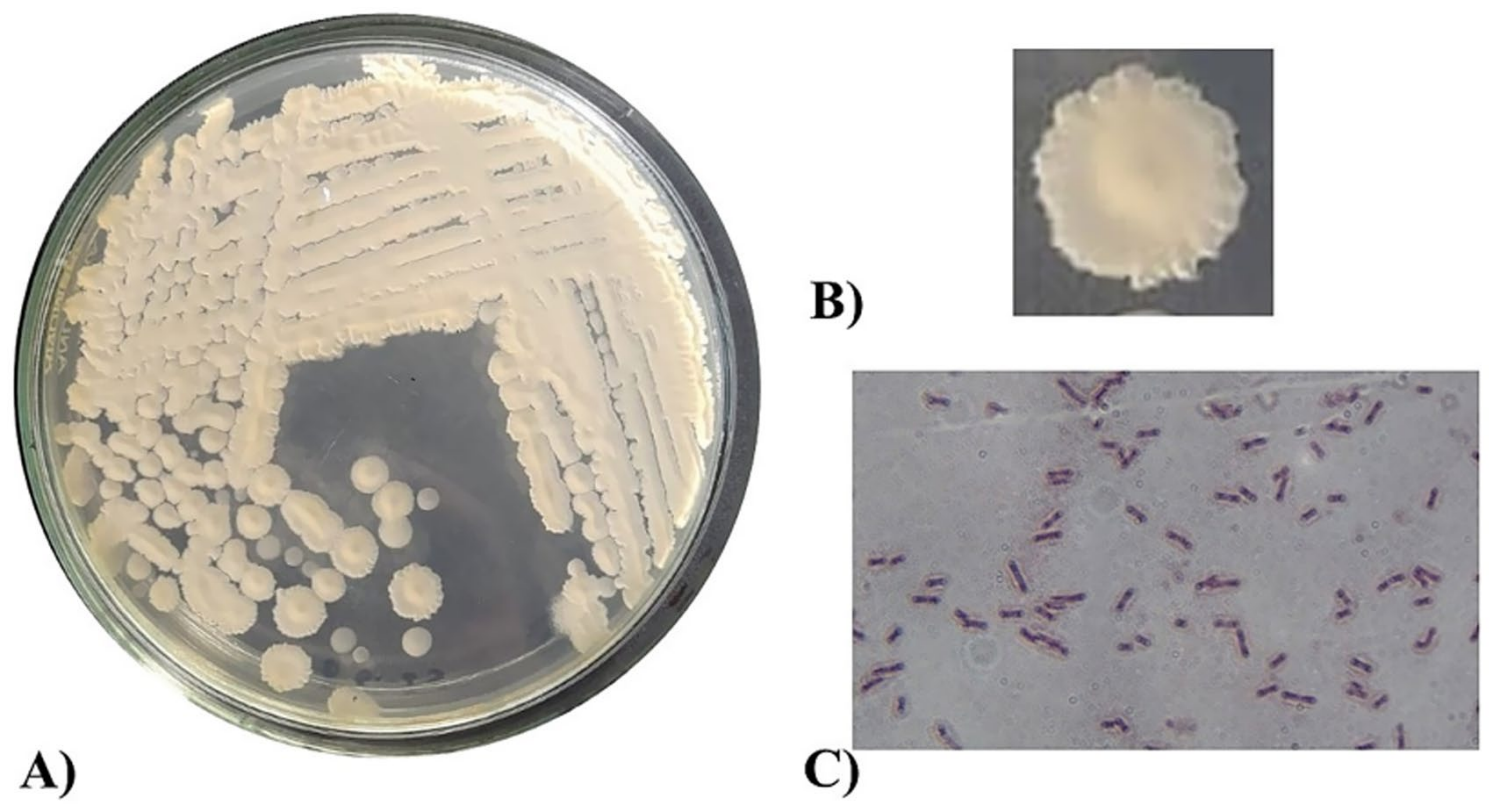
**(A)** Pure culture of F2. **(B)** Morphological characteristics of a single colony of strain F2. **(C)** Microscopic image of single cells. It shows the rhizoid-shaped colony of isolate F2 with undulate margins. The microscopic image shows the cocci-shaped cells forming long chains.

**Figure 4:**
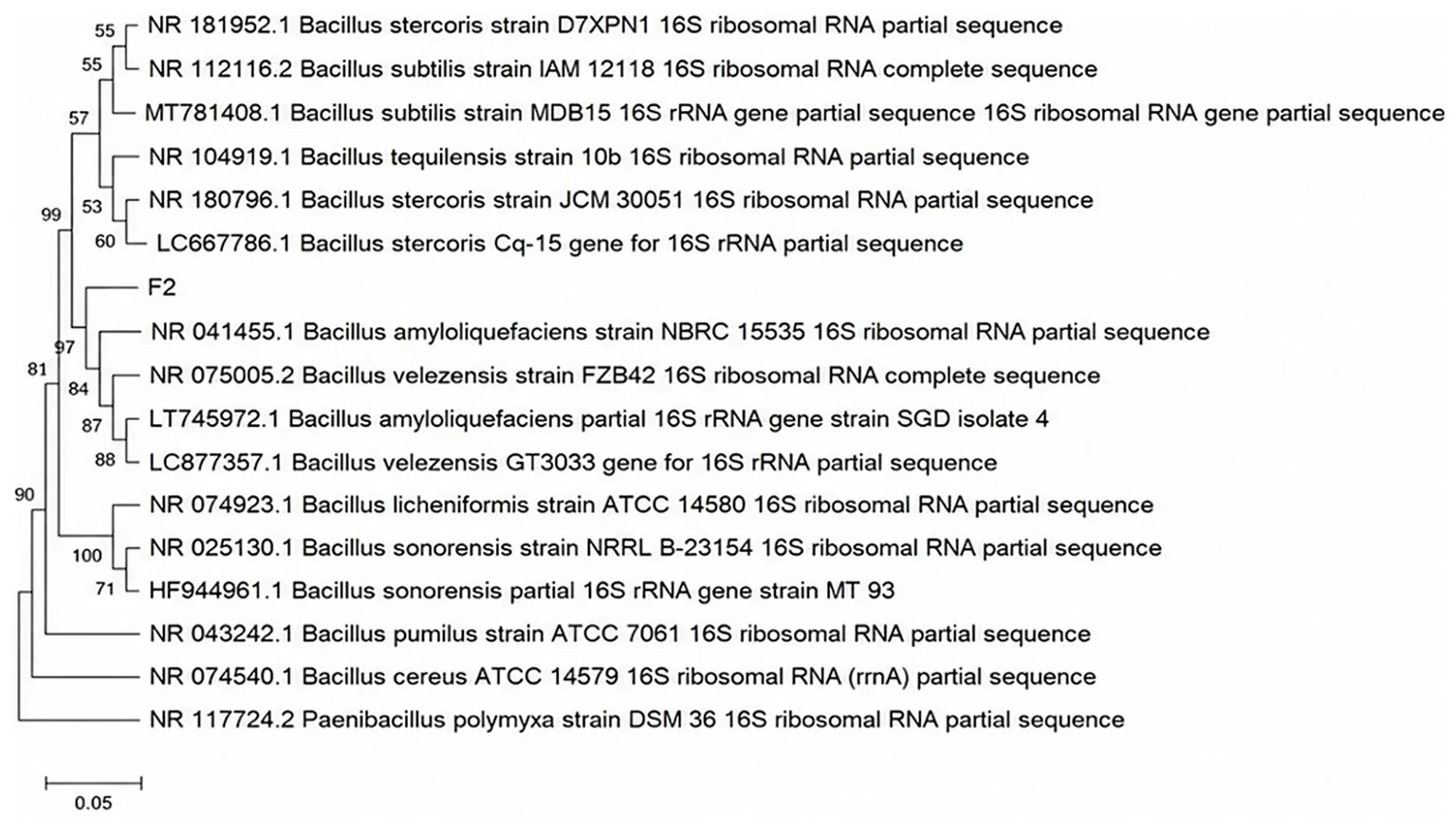
The 16S rRNA gene-based phylogenetic tree was constructed in MEGA version 11.0.13 using the Neighbor-Joining method with 1,000 bootstrap values. This analysis includes the sequences of the bacterial isolate F2 and the closely related *Bacillus* spp. (sequences retrieved from the NCBI database) and *Paenibacillus polymyxa* strain DSM 36, which was used as an outgroup. The isolate was clustered within the *Bacillus amyloliquefaciens* clade, showing close genetic association with the *Bacillus amyloliquefaciens* strain NBRC, with a strong bootstrap value of 97%.

### 3.3 Biochemical characterization

The biochemical assays indicated that isolate F2 was positive for IAA, siderophore, and ammonia production but was negative for HCN production. It did not show any solubilization activity for potassium, phosphate, and zinc minerals (Table 4; Figure 5). F2 was found to be the active producer of amylase, pectinase, protease, chitinase, cellulase, and catalase enzymes. It was negative for esterase and lipase activity (Table 4; Figure 6).

**Table 4:** Biochemical characteristics of bacterial isolate F2.

Bacterial isolate	PGPR activities						
	Indole acetic acid	HCN production	Siderophore production	Ammonia Production	Potassium solubilization	Phosphate solubilization	Zinc solubilization
F2	+	−	+	+	−	−	−

	Hydrolytic enzyme assay							
	Amylase	Protease	Pectinase	Catalase	Lipase	Cellulase	Chitinase	Esterase
F2	+	+	+	+	−	+	+	−

**Figure 5:**
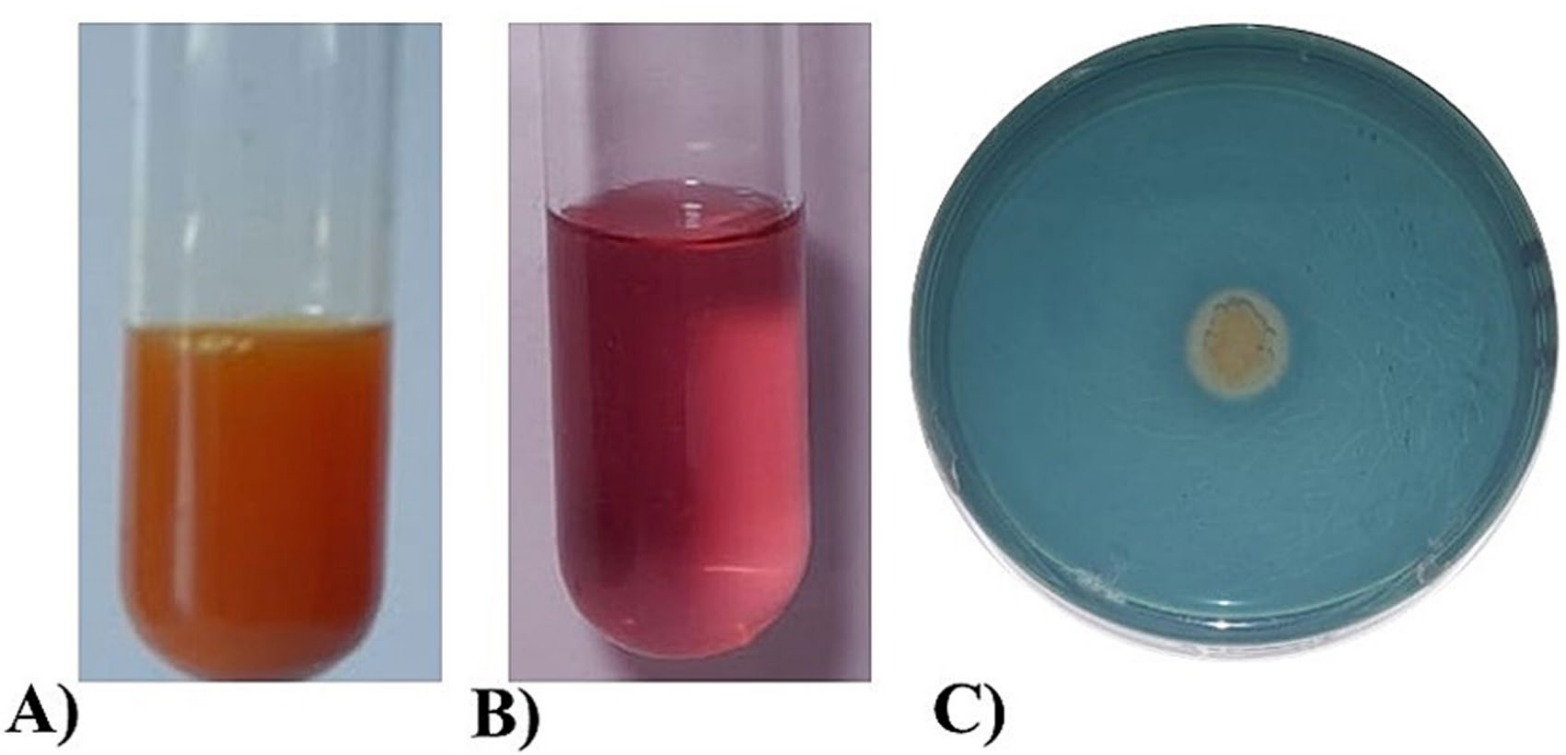
PGPR activities of bacterial isolate F2. **(A)** Ammonia production activity indicated by development of orange color upon addition of Nessler’s reagent. **(B)** IAA production activity indicated by development of pink color upon addition of Salkowski reagent. **(C)** Positive siderophore production indicated by formation of a halo zone in CAS agar.

**Figure 6:**
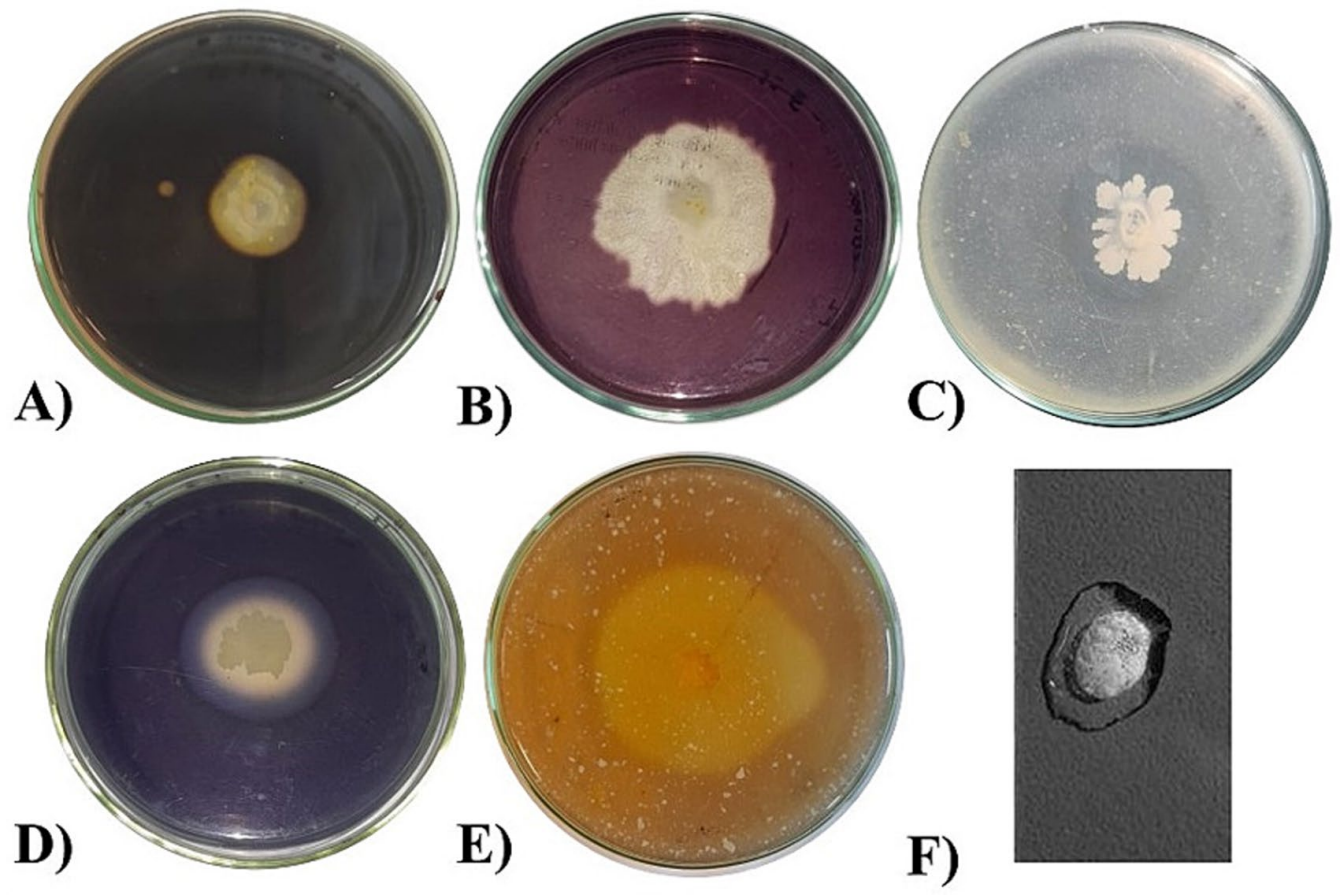
Hydrolytic enzyme assays of bacterial isolate F2. **(A)** Amylase production indicated by development of a halo zone after staining with Lugol’s iodine; **(B)** Cellulase production indicated by development of a halo zone after staining with Lugol’s Iodine; **(C)** Protease production indicated by halo zone formation in skimmed milk agar plates; **(D)** Pectinase production indicated by halo zone after staining with Lugol’s iodine; **(E)** Chitinase production indicated by development of halo zone after staining with Lugol’s iodine; **(F)** Catalase production indicated by bubble formation upon addition of Hydrogen peroxide.

### 3.4 PCR amplification of biocontrol-related genes

Biocontrol-associated gene screening showed that all seven genes, *srfAA*, *fend*, *ituC*, *yngG*, *yndJ*, *yndJ*, and *dhbA*, are responsible for the synthesis of non-ribosomal peptide synthetase (NRPS) metabolites; all four genes, *mln*, *bae*, *dfn*, and *bac*, are responsible for biosynthesis of polyketide synthetase (PKS) metabolites and all two genes; *sboA* and *bioA*, are responsible for biosynthesis of RPS were found in F2 bacterial isolate (Table 5; Figure 7).

**Table 5:** Biocontrol-related gene profile of bacterial isolate F2.

Biocontrol-related genes		Bacterial isolate
		F2
Non-ribosomal peptide synthetases (NRPS)	*srfAA*	+
	*fenD*	+
	*yndJ*	+
	*bamD*	+
	*ituC*	+
	*dhbA*	+
	*yngG*	+
Polyketide synthetases (PKS)	*mln*	+
	*bae*	+
	*dfn*	+
	*bac*	+
Ribosomal peptide synthetases (RPS)	*sboA*	+
	*bioA*	+

**Figure 7:**
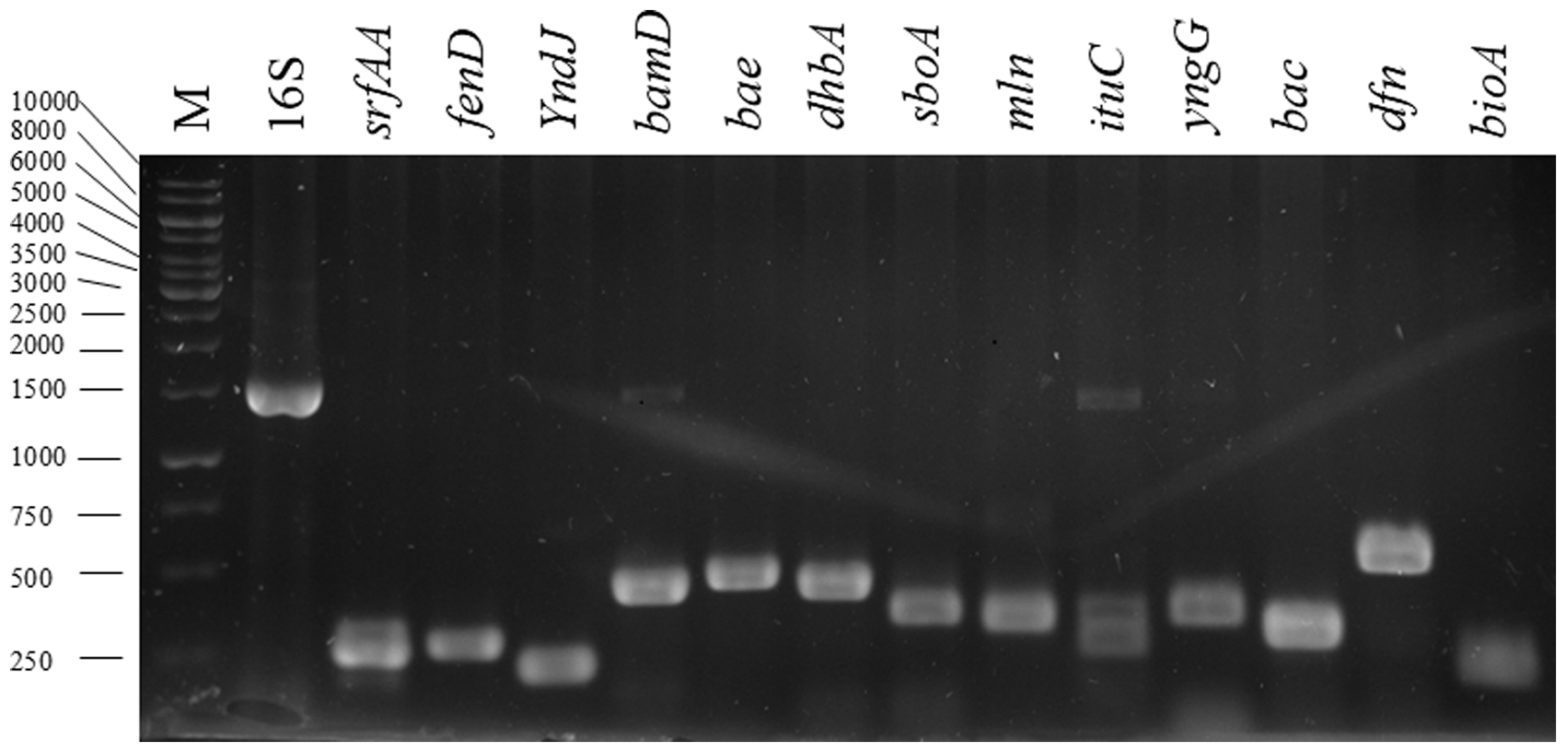
Agarose gel electrophoresis showing PCR amplification of biocontrol-related genes using genomic DNA of bacterial isolate F2. M indicates the DNA molecular weight marker. A 1 kb ladder was used. The gene names are labeled on the gel image.

### 3.5 Expression analysis of biocontrol-related genes

Co-cultivation of bacterial isolate F2 with the fungus, *F. Oxysporum* f. sp. *cubense* TR4, resulted in the significant up-regulation of biocontrol-related genes as compared with bacterial isolate F2 grown alone. The highest level of up-regulation was observed for *fenD* (30.83 ± 4.7), *srfAA* (27.64 ± 2.32), *ituC* (26.33 ± 3.98), and *dhbA* (21.94 ± 3.78) (Figure 8).

**Figure 8:**
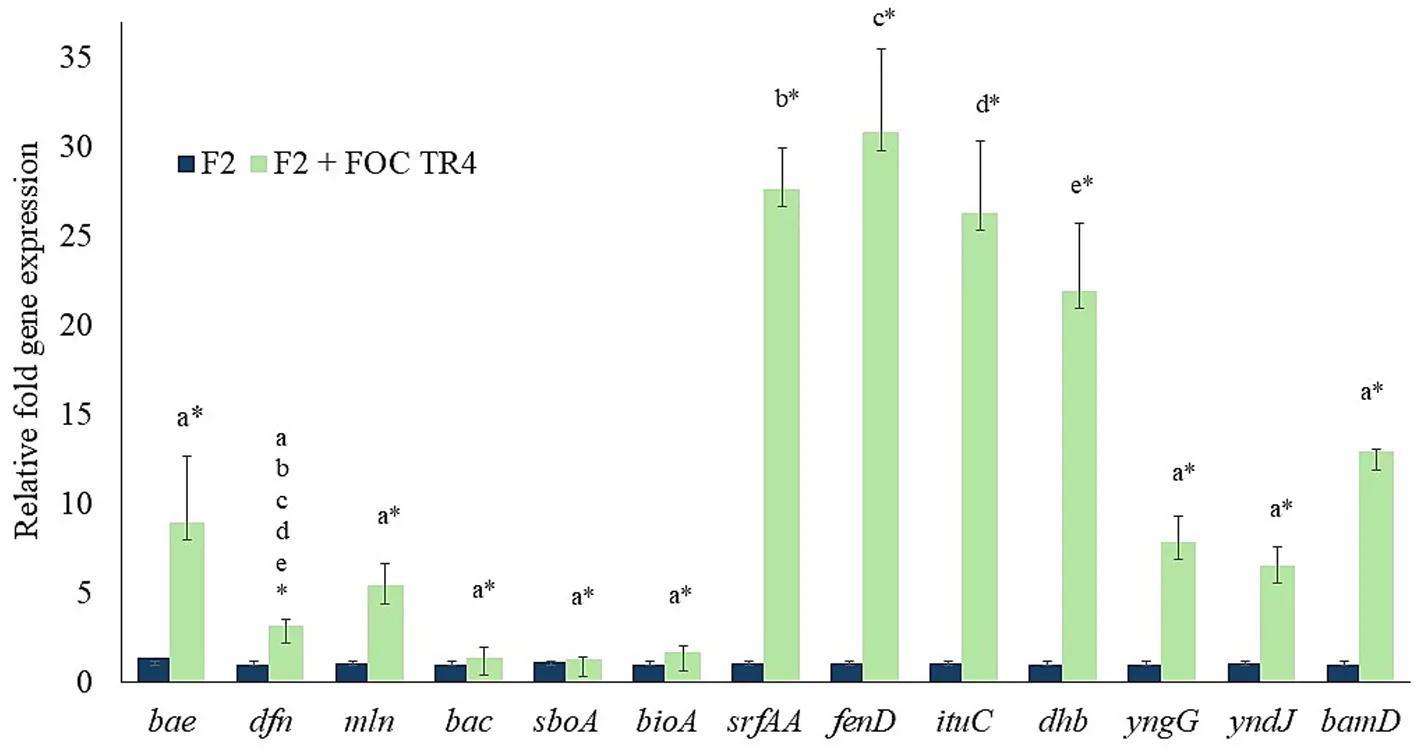
Expression profile of the biocontrol genes (mean value ± SE) in F2 and F2 + Foc TR4. The asterisk represents a significant difference in gene expression in F2 + *FOC TR4.* The different lowercase letters show significant differences in gene expression among all the genes. All the genes were upregulated in the interaction samples except for *bac*, *sboA*, and *bioA*. Among all the genes, *fenD* was highly upregulated, followed by *srfAA*, *ituC*, *dhb*, and *bamD*.

### 3.6 *In vitro* analysis of aseptic filtrate

The cell-free filtrate of F2 showed antagonistic activity against all fungal isolates. It inhibited the growth of fungus *in vitro* by 38% on average, ranging from 31 to 48% (Table 6; Figure 9).

**Table 6:** Percentage mycelial inhibition of Foc TR4 isolates by the aseptic filtrate of endophytic bacterial isolate F2.

Sr. no	Fungal isolate	Aseptic filtrate of F2
1	P.TH4-L1-DAS	32.15 ± 1.6
2	P.TH5-L2-DAS	35.41 ± 1.8
3	P.TH6-L3-DAS	41.66 ± 2.06
4	P.TH7-L4-DAS	32.38 ± 1.64
5	P.TH8-L5-DAS	38.21 ± 1.40
6	P.TH9-L6-DAS	34.23 ± 1.56
7	P.TH10-L7-MF	24.50 ± 2.13
8	P.TH11-L8-MF	34.23 ± 1.56
9	P.TH12-L9-DAS	48.33 ± 1.45
10	P.TH13-L10-103MN	36.09 ± 2.09
11	P.TH14-L11-103MN	39.87 ± 2.62
12	P.TH15-L12-103MN	32.59 ± 1.28
13	P.TH16-L13-103MN	31.74 ± 1.37
14	P.TH17-L14-103MN	21.66 ± 1.44
15	P.TH18-L15-103MN	40.90 ± 3.93
16	P.TH19-L16-BPD	44.16 ± 1.44
17	P.TH20-L17-BPD	42.98 ± 3.04
18	P.TJ1-L18-KF	31.33 ± 1.44
19	P.TJ2-L19-KF	32.98 ± 3.03
20	P.TJ3-L20-KF	39.28 ± 1.34
21	P.NS1-L21-SK	48.33 ± 1.45

**Figure 9:**
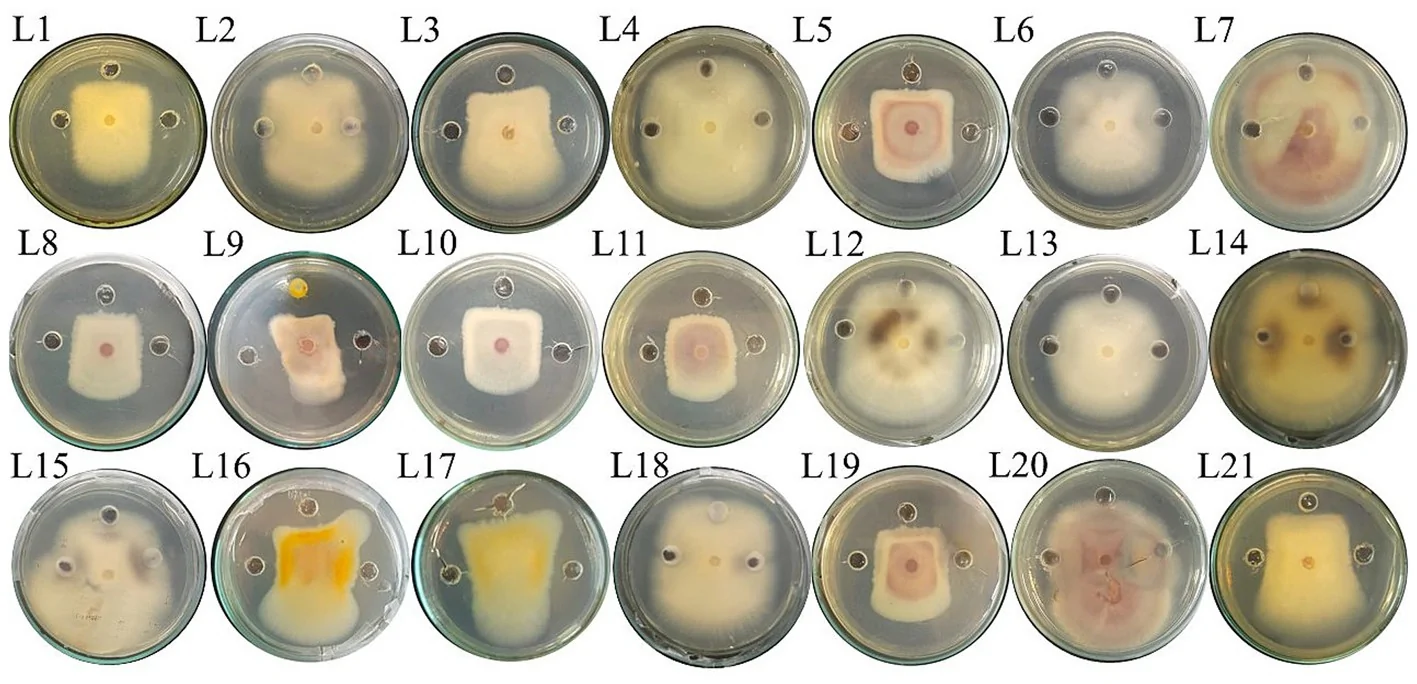
*In vitro* evaluation of antagonistic activity of aseptic filtrate of endophytic bacterial strain F2 against 21 isolates of *F. oxysporum* f. sp. *cubense.* It shows the varying degree of inhibition by the aseptic filtrate toward each fungal isolate, ranging from very weak to moderate and high levels of inhibition.

### 3.7 Gas chromatography–mass spectrometry (GC–MS)

GC–MS profiling of the cell-free extract of all three groups detected different compounds. A total of 25 compounds were identified from FOC TR4 extract, 17 from F2 extract, and 73 from the interaction of F2 and FOC TR4. The FOC TR4 extract contained diketopiperazine compounds (15.6%), ketones and their derivatives (2.3%), carboxylic acids and esters (15.6%), amides (3.2%), and pyranones (6.9%). The F2 extract contained ketones (1.73%), hydrocarbons (1.69%), diketopiperazines (0.58%), ether derivatives (0.57%), phenolic compounds (0.36%), and fatty acid methyl esters (0.28%). The extract of co-culture F2 and FOC TR4 was composed of Diketopiperazines (29%), dipeptide derivatives (16%), carboxylic acids and their derivatives (13%), organic acids and fatty acids (13%), phenolic compounds (6%), amino acids and their derivatives (30%), nitrogen-containing heterocyclic compounds (7%), amides (11%), amines (13%), carboxylic acids and esters (7%), alkaloids (0.5%), lactams (10%), and hydrocarbons (5%). Of the identified compounds, 36 distinct compounds were detected in the F2 and FOC TR4 interaction extract. Most of these compounds are produced by the bacterial isolate to inhibit pathogenic fungi, while some of them are produced in response to stress (Table 7; Figures 10A–C).

**Table 7:** Compounds detected upon GC–MS analysis of crude extracts of Foc TR4, F2, and F2 + Foc TR4.

Sample	Metabolites	t_R_(min)	Peak area (%)
Foc TR4	2-Propanone	2.159	2.39
	1-(1,3-dioxolan-2-yl)	2.159	2.39
	Acetic acid	2.159	2.39
	Butanoic acid	2.615	1.80
	4-iodo-	2.615	1.80
	methyl ester	2.615	1.80
	4-hydroxy-4-methyl- Silane	2.615	1.80
	trimethyl-	2.615	1.80
	4H-Pyran-4-one	6.877	1.66
	2,3-dihydro-3,5-di hydroxy-6-methyl-	6.877	1.66
	2,4-Dihydroxy-2,5-dimethyl-3(2H)-furan-3one	6.877	1.66
	Pyrrolo[1,2-a]pyrazine-1,4-dione	15.022	9.74
	hexahydro-	15.022	9.74
	*3,6-bis(2-methylpropyl)-*	18.995	5.87
	DL-Norleucine	18.995	5.87
	N-allyloxycarbonyl-	18.995	5.87
	allyl ester	18.995	5.87
	nonyl ester	19.132	3.75
	Butyramide	19.132	3.75
	N-allyl-N-hexyl-2-bromo	19.132	3.75
	1,3-benzenedicarboxaldehyde	19.132	3.75
	4-hyd roxy-5,6-dimethoxy-	19.132	3.75
	Biacetyl mono((3,4-dimethyl-5-isox azolyl)hydrazone) monoxime	19.132	3.75
	9-Octadecenamide (Z)-	20.631	3.15
	Bis(2-ethylhexyl) phthalate	22.025	71.63
F2	2-Pentanone	2.619	1.73
	4-hydroxy-4-methyl-	2.619	1.73
	2,4-Di-tert-butylphenol	12.115	0.36
	Pyrrolo[1,2-a]pyrazine-1,4-dione	14.946	0.58
	hexahydro-	14.946	0.58
	Heptadecanoic acid	18.642	0.28
	16-methyl-	18.642	0.28
	methyl ester	18.642	0.28
	Methyl stearate	18.642	0.28
	2-[2-[2-[2-[2-[2-[2-[2-[2-[2-(2-Methoxyethoxy)ethoxy]ethoxy]ethoxy]ethoxy]ethoxy]ethoxy] ethoxy]ethoxy]ethoxy]ethanol	18.831	0.57
	D-(+)-Arabitol	18.831	0.57
	pentamethyl ether	18.831	0.57
	1,4,7,10,13,16-Hexaoxacyclooctadecane	18.831	0.57
	Cyclopentane	19.103	1.69
	1,2,4-trimethyl-	19.103	1.69
	(1.alpha.,2.beta.,4.alpha.)- 6,6-Dimethyl-cyclohex-2-en-1-ol	19.103	1.69
	Bis(2-ethylhexyl) phthalate	22.031	94.54
F2 + Foc TR4	Propanoic acid	2.229	7.63
	2-methyl-	2.229	7.63
	Butanoic acid	2.769	5.92
	Pentanoic acid	2.769	5.92
	Octanoic acid	2.769	5.92
	2,4-Di-tert-butylphenol	12.116	0.41
	2,5-Piperazinedione	13.105	0.69
	3-methyl-	13.105	0.69
	2-Pyrrolidinone*	13.105	0.69
	3-Methyl-2,3,6,7,8,8a-hexahydropyrrolo[1,2-a]pyrazine-1,4-dione	14.475	1.80
	3-Pyrrolidin-2-yl-propionic acid*	14.475	1.80
	Azetidine	14.475	1.80
	1,1′-methylenebis-	14.475	1.80
	3-Methyl-1,4-diazabicyclonan-2,5-dione*	14.762	1.53
	N-acetyl- Azetidine*	14.762	1.53
	Pyrrolo[1,2-a]pyrazine-1,4-dione	15.132	26.12
	Hexahydro	15.132	26.12
	Cyclo(L-prolyl-L-valine)*	15.575	0.48
	Hexahydro-3-(1-methylpropyl)pyrrolo[1,2-a]pyrazine-1,4-dione*	15.575	0.48
	Phenol	15.575	0.96
	3,5-dimethoxy	15.575	0.96
	2-Ethyl-1,3,4-trimethyl-3-pyrazolin-5-one	15.866	0.96
	hexahydro-3-(2-methylpropyl)-*	16.718	0.78
	Sebacic acid*	16.718	0.78
	2,6-dimethoxyphenyl tridecyl ester*	16.718	0.78
	L-Valine*	16.868	0.42
	n-propargyloxycarbonyl-*	16.868	0.42
	heptadecyl ester*	16.868	0.42
	l-Proline	16.868	0.42
	N-allyloxycarbonyl-	16.868	0.42
	heptyl ester	16.868	0.42
	2-Piperidinone*	17.274	9.66
	1-methyl-*	17.274	9.66
	Methanamine*	17.274	9.66
	bis(2-trifluoroacetoxyethyl)-*	17.274	9.66
	Oxalic acid*	17.274	9.66
	Diamide*	17.274	9.66
	N, N, N′, N′-tetrahexyl-*	17.274	9.66
	Piperidine	17.535	3.93
	3,5-dimethyl-	17.535	3.93
	1,1’-Biphenyl	17.535	3.93
	4-methoxy-*	17.535	3.93
	N, N′-di-n-Butyl-1,6-hexanediamine*	17.535	3.93
	Methyl stearate	18.642	1.00
	o-Hydroxybiphenyl*	18.831	1.20
	l-Norleucine,	18.831	1.20
	hexadecyl ester	18.831	1.20
	l-Leucine	18.831	1.20
	2,5-Piperazinedione	19.057	7.74
	3,6-bis(2-methylpropyl)-	19.057	7.74
	3-Cyclopentylpropionic acid*	19.057	7.74
	4-biphenyl ester*	19.057	7.74
	N-butoxycarbonyl-	19.057	7.74
	hexyl ester	19.057	7.74
	l-Prolylglycine*	19.139	16.17
	methyl ester	19.139	16.17
	d-Proline	19.139	16.17
	N-isobutoxycarbonyl-	19.139	16.17
	sobutyl ester*	19.139	16.17
	propyl ester*	19.139	16.17
	Pyrrolo[1,2-a]pyrazine-1,4-dione	20.353	0.54
	hexahydro-3-(phenylmethyl)-*	20.353	0.54
	Ergotaman-3′,6′,18-trione*	20.353	0.54
	9,10-dihydro-12′-hydroxy-2′-methyl-5′-(phenylmethyl)-*	20.353	0.54
	(5′.alpha.,10.alpha.)-*	20.353	0.54
	Bis(2-ethylhexyl) phthalate	21.998	7.67
	L-Phenylalanine*	22.314	1.82
	N-(2,6-difluorobenzoyl)-*	22.314	1.82
	Benzamide*	22.314	1.82
	2,4-difluoro-N-methyl-N -butyl- Barbituric acid*	22.314	1.82
	5,5-dipropyl-*	22.314	1.82
	4,5,6,6a,6b,7,8,12b-Octahydrobenzo (J)fluoranthene*	22.314	1.82
	2,6-difluoro-N-(2-butyl)-N-ethyl-*	22.314	1.82

The asterisk shows the distinct compound present in the F2 and FOC TR4 interaction sample.

**Figure 10:**
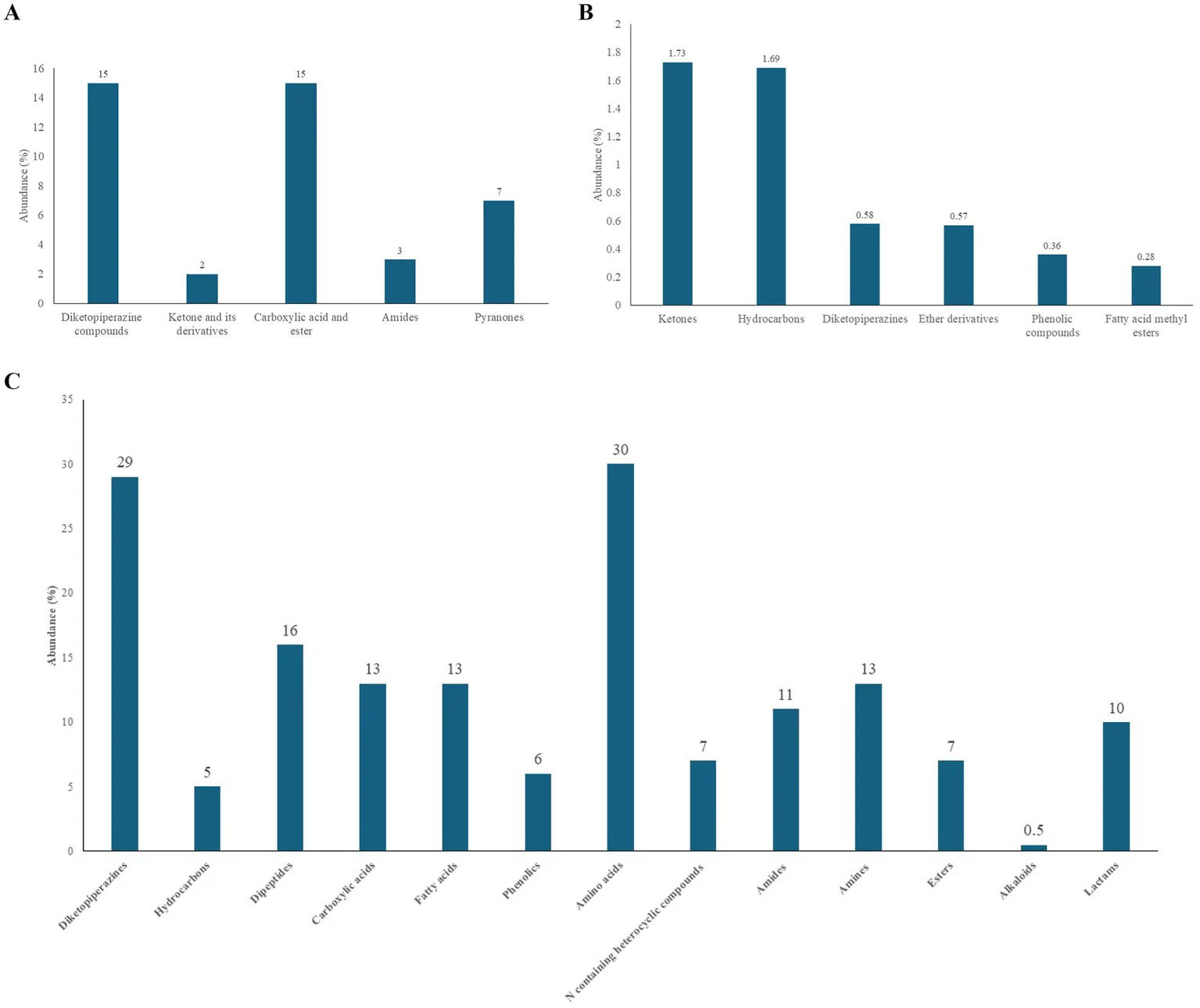
**(A)** Relative abundance of compounds identified by GC–MS analysis of the Foc TR4 extract. This graph shows that the Diketopiperazine compounds, carboxylic acids, and esters are found in abundance in the extract, followed by Pyranones and low amounts of Amides and Ketones and their derivatives. **(B)** Relative abundance of compounds identified by GC–MS analysis of F2 extract. This graph shows the abundance of Ketones and Hydrocarbons in the extract and traces of Diketopiperazines, Phenolics, ethers, and Fatty acids. **(C)** Relative abundance of compounds identified by GC–MS analysis in the extract of F2 + Foc TR4. This graph indicates that Amino acids and Diketopiperazines are in abundance, and moderate amounts of Dipeptides, Carboxylic acids, Amides, Amines, Lactams, low amounts of hydrocarbons, esters, phenolics, nitrogen-containing cyclic compounds, and traces of Alkaloids are present in the extract.

## 4 Discussion

A total of 138 bacteria were isolated from the soil, roots, leaves, and midrib tissues of healthy banana plants and screened for their biocontrol potential against 21 genetically diverse isolates of *Fusarium oxysporum* f. sp. *cubense* Tropical Race 4 (Foc TR4). The study results revealed that the isolated bacteria exhibited varying degrees of mycelial growth inhibition. Among the rhizosphere-, root-, and stem-derived bacterial isolates, 7 showed strong antagonistic activity. Bacterial isolate F2, isolated from the rhizosphere of healthy banana plants, showed strong antagonistic activity against all 19 Foc TR4 fungal isolates, and the activity ranged from 65 to 74%. Molecular characterization indicated that this isolate showed a close similarity to *Bacillus amyloliquefaciens* strain S1.

*Bacillus* spp. is a highly diverse group of bacteria. They are rich in traits like stress tolerance, broad-spectrum antibacterial activity, eco-friendly nature, plant growth-promoting traits, and a highly adaptive nature (Cheng et al., 2019). It is marked as an important taxon as it plays important physiological roles in the endospheric microbiota of many plant species like rice, olive, citrus, and banana (Ali, 2026). Among all the antagonistic strains of *Bacillus* spp., *Bacillus amyloliquefaciens* is marked as an important antagonist to plant pathogens, as it suppresses pathogens via competition for nutrients, production of antibiotics and secondary metabolites, and induction of systemic resistance in plants (Cruz-Martín et al., 2021).

*Bacillus amyloliquefaciens* has been successfully used to control plant diseases caused by foliar and soil phytopathogens. Wu et al. (2014) demonstrated the antagonistic efficiency of *B. amyloliquefaciens* NJZJSB3 strain in controlling *F. oxysporum* f. sp. *cucumerinum* (70%), *F. oxysporum* f. sp. *niveum* (66.4%), *F. oxysporum* f. sp. *cubense* (63.8%), and *F. oxysporum* f. sp. *dioscorea* (46%). Similarly, *B. amyloliquefaciens* strains showed antagonistic activity against strawberry anthracnose, *Fusarium oxysporum* f. sp. *spinaciae*, and *F. oxysporum* f. sp. *niveum* (Es-Soufi et al., 2020; Zhao et al., 2014, 2018). Furthermore, 40% growth inhibition of *F. oxysporum* f. sp. *cubense* was reported using *Bacillus amyloliquefaciens* NJN-6, isolated from the rhizosphere soil of healthy banana plants (Yuan et al., 2012). Similarly, a novel biofertilizer consisting of fermented *Bacillus amyloliquefaciens* strain NJN-6 has been shown to exhibit an excellent disease suppression ability against banana Fusarium wilt disease based on one seasonal pot experiment (Zhang et al., 2014). More recently, (Fan et al., 2021) reported that a *B. amyloliquefaciens* strain isolated from healthy banana rhizosphere soil inhibited Foc TR4 by 79.6% *in vitro* and reduced disease severity by 82.6% under field conditions. In the current study, 60% *in vitro* inhibition by the F2 bacterial isolate falls within the lower-to-middle range of these reported values (Yuan et al., 2012; Wu et al., 2014; Fan et al., 2021). This is significant because it demonstrates that F2 possesses *in vitro* biocontrol capacity comparable to other rhizosphere-associated *B. amyloliquefaciens* strains already established as effective agents against Foc TR4. Further *in vivo* validation of its biocontrol efficacy would strengthen its potential as a biocontrol agent.

Different strains of *Bacillus amyloliquefaciens* are known to suppress Foc TR4 by different yet overlapping mechanisms. These include structural deformities in fungal hyphae, fungal cell wall breakdown, and inhibition of spore germination by production of secondary metabolites, antibiotics, and volatile organic compounds (Sudharsan et al., 2026).

Following the establishment of the biocontrol potential of the F2 bacterial isolate, the isolate was further characterized for various plant growth-promoting and biocontrol-associated traits. These included plant growth-promoting characteristics, production of hydrolytic enzymes, production of siderophores, production of HCN, presence and expression of biocontrol genes, and production of VOCs. The results showed that the F2 bacterium possesses PGPR activities in addition to antagonistic activity toward Foc TR4. It actively produces IAA, siderophores, and ammonia but is negative for HCN production and phosphate, potassium, and zinc solubilization. Despite lacking these classical PGPR traits, F2 displayed the highest antifungal activity, suggesting that effective suppression of FocTR4 may depend more strongly on antimicrobial metabolite production and antagonistic interactions than on nutrient solubilization alone. IAA is a plant hormone that promotes root and shoot growth and also induces systemic resistance in plants against phytopathogens (Fu et al., 2015). Siderophores act as iron chelators, create competition for iron in the environment, and make the pathogen starve for it (Kashyap et al., 2021; Deb and Tatung, 2024). Ammonia production creates an alkaline environment that inhibits spore germination; on the other hand, it provides biofertilizer for the soil to promote plant growth (Zheng et al., 2012; Abo-Zaid et al., 2020). Our findings were in agreement with the findings of Fan et al. (2021), who reported that a *B. amyloliquefaciens* strain isolated from healthy banana rhizosphere soil inhibited Foc TR4 and also promoted banana plant growth.

Hydrolytic enzyme profiling of the F2 bacteria further supported its antagonistic capacity against Foc TR4. Our results showed that F2 was positive for the production of amylase, pectinase, chitinase, catalase, cellulase, and protease. Rhizosphere-associated *Bacillus* species are capable of producing enzymes such as chitinases, cellulases, Glucanases, proteases, and lipases, which may contribute to suppression of fungal pathogen growth (Majeed et al., 2015; Ayyaz et al., 2021). *B. stercoris*, *B. subtilis*, and *B. halotolerans* isolated from the rhizosphere were reported to produce different levels of chitinase, cellulase, pectinase, and protease, highlighting the importance of extracellular enzymes in the antagonistic activity of rhizobacteria (Aslam et al., 2025). *B. macquariensis*, *B. macerans*, and *B. circulans* isolated from the tomato rhizosphere demonstrated the production of hydrolytic enzymes, including chitinase, glucanase, protease, and cellulase (Ayantola and Fagbohun, 2020). These extracellular enzymes are considered important traits of rhizobacterial biocontrol agents and may contribute to the suppression of fungal pathogens. In particular, chitinase can hydrolyze chitin, a major structural component of the cell walls of fungi, whereas other hydrolytic enzymes, including glucanases and proteases, may further contribute to the degradation of fungal cell wall components (Ajuna et al., 2023). The production of multiple hydrolytic enzymes by F2 may therefore represent a potential mechanism underlying its strong antagonistic activity.

Molecular analysis using biocontrol genes provides evidence that antimicrobial secondary metabolism may contribute significantly to the antagonistic activity of the F2 bacterial isolate. Upregulation of seven genes (*bae*, *mln*, *srfAA*, *fenD*, *ituC*, *dhbA*, and *BamD*) during F2 interaction with Foc TR4 compared with F2 grown without pathogen exposure was observed in the current study. High expression of these genes during F2-FocTR4 interaction suggests that in F2 bacteria these genes were activated upon interaction with FocTR4. Upregulation of *srfAA*, *fenD*, *and ituC, involved in the biosynthesis of surfactin, fengycin, and iturin, respectively, showed potential involvement of cyclic lipopeptides in the antagonistic activity of F2 against* FocTR4. LC–MS-based analysis would further help to validate the production and quantification of these lipopeptides during the interaction. However, there are a number of studies that have shown that the production of cyclic lipopeptides is considered an important mechanism involved in the antagonistic activity exhibited by Bacillus species against soil-borne pathogens. Surfactin, fengycin, and Iturin are well-characterized metabolites that can inhibit fungal growth by disrupting fungal membrane integrity, inhibiting fungal membrane development, transcription of immune defense-related genes, and production of reactive oxygen species. Fengycin, produced by *Bacilli* bacteria, induces cell death by cell membrane attraction and by increasing cell permeability, which results in ultrastructural destruction of fungal structures such as hyphae and production of Reactive oxygen species (ROS), which leads to breakdown of cell membranes and ultimately cell death (Markelova and Chumak, 2025). Surfactin plays an important role in the inhibition of Foc TR4 by initiating antagonistic mechanisms and also by upregulating the biosynthesis of plant defense genes (Ruan et al., 2024). Surfactin in combination with fengycin effectively inhibits the fungal pathogen. Bacillomycin D and fengycin produced by *B. amyloliquefaciens* SQR9 collectively inhibit Fusarium wilt effectively (Xu et al., 2013). *Bacillus amyloliquefaciens* has also been reported to be highly effective in controlling various phytopathogens through the production of lipopeptide antibiotics. The cell-free extract of *B. amyloliquefaciens* LYZ69 contained bacillomycin D, fengycin A, and fengycin B. Bacillomycin D has been confirmed to inhibit the growth of fungi such as *Fusarium graminearum* (Gu et al., 2017). Moreover, LYZ69 produces diverse cyclic lipopeptides that can enhance the antifungal spectrum of the strain (Liu et al., 2020). A study by Hu et al. (2021) demonstrates the efficacy of LYZ69 against Anthracnose of Alfalfa by producing cyclic lipopeptides, under greenhouse conditions. In another study, a strain isolated from the rhizosphere of a healthy banana plant, identified as *Bacillus amyloliquefaciens*, inhibited Foc TR4 by 79.6% *in vitro*. In field trials, it suppressed the disease by 82.6% and promoted the growth of banana plantlets by increasing the biosynthesis of antibiotics (Fan et al., 2021).

In addition to genes associated with antimicrobial metabolites production, high expression of *dhbA* provide further evidence for the role of iron competition in antagonistic activity of F2 bacterial isolate. This gene is involved in the biosynthesis of bacillibactin, a siderophore involved in Iron chelation. This finding supports the possibility of siderophore-mediated competition and contributes to the overall biocontrol potential of F2 bacteria alongside other mechanisms.

The upregulation of these genes associated with different biocontrol functions suggests that the antagonistic activity of the F2 bacterial isolate does not depend on a single compound and may involve multiple complementary mechanisms. Such mechanisms have already been identified in rhizosphere-associated *Bacillus* strains capable of suppressing Fusarium wilt (Fan et al., 2021; Gu et al., 2017; Xu et al., 2013).

The antagonistic potential of F2 is further supported by GC–MS analysis, which demonstrated that the volatile organic compound and metabolite profile of the bacterium changed following interaction with Foc TR4. Several compounds were detected specifically or at increased abundance in the interaction treatment. These changes may reflect the metabolic response of F2 to interaction with the Foc TR4 isolate. However, since the initial profiling of F2 bacteria was performed in LB medium and Foc TR4 was grown in PDB, whereas the profiling of the F2-Foc TR4 interaction was performed when F2 bacteria were growing in PDB, the change in the culture medium for F2 bacteria could also contribute to this increase in abundance in the interaction study. These compounds included diketopiperazines (DKPs), amino acid derivatives, fatty acids, organic acids, benzamide derivatives, and phenolic compounds. The elevation of these metabolites during F2-Foc TR4 interaction demonstrates that F2 responds metabolically to the pathogen and activates specific chemical defenses. Similar metabolite classes have previously been detected in *Bacillus* spp. exhibiting antagonistic activity against Foc TR4 (Muthulakshmi et al., 2019). Specifically, compounds in the DKP class and fatty acids such as octanoic acid and pentanoic acid have been associated with antimicrobial activity. Additionally, 2,4-di-tert-butylphenol has been reported to possess antifungal activity by affecting fungal membranes and inhibiting spore germination (Nayana R U et al., 2025; Stocki et al., 2025). Benzoic acid derivatives may also contribute to antifungal activity and plant defense-associated processes (Alsalmo et al., 2026; Suparmin et al., 2026). Several rhizosphere-associated *Bacillus* isolates have previously been reported to produce VOCs with inhibitory activity against *Fusarium* species. VOC compounds produced by *Bacillus* spp. operate through mechanisms distinct from direct physical contact and can suppress fungal development by inhibiting mycelial growth, spore germination, and fungal gene expression over distance (Stocki et al., 2025). The VOC-mediated inhibition documented here provides an additional explanation for the strong antagonistic activity of F2 bacterial isolate against Foc TR4. *Bacillus amyloliquefaciens* strains isolated from the rhizosphere have been shown to produce volatile compounds that suppress the growth and development of *Fusarium oxysporum* (Yuan et al., 2012; Wu et al., 2019).

The findings of the current study suggest that the rhizosphere-associated F2 isolate showed antagonist activity through multiple mechanisms, such as the production of hydrolytic enzymes, secondary metabolites belonging to classes named polyketide synthetases, Ribosomal peptide synthetases, and non-ribosomal peptide synthetases, and production of volatile organic compounds against Foc TR4 rather than relying on a single mode of action.

## 5 Conclusion

Screening of 138 healthy banana plant-associated bacterial isolates showed 7 bacterial isolates with strong antagonistic activity. However, among these isolates, the F2 bacterial isolate associated with the rhizosphere of the Banana plant demonstrated significantly high mycelial growth inhibition against 19 Foc TR4 fungal isolates. F2 isolate was identified as *Bacillus amyloliquefaciens*. The presence and up-regulation of biocontrol-associated genes, antagonistic activity of cell-free filtrate, and change in metabolomic profiling during interaction with Foc TR4 suggest that F2 may suppress mycelial growth through multiple mechanisms. These findings present F2 bacterial isolate as a promising candidate for biocontrol agent against Banana Fusarium wilt disease.

## Data Availability

The datasets presented in this study can be found in online repositories. The names of the repository/repositories and accession number(s) can be found in the article/Supplementary material.

## Footnotes

1
http://www.dna-dragon.com/
