# Supplementary material for: Biocontrol potential of a rhizosphere-associated Bacillus amyloliquefaciens F2 isolate against Fusarium oxysporum f. sp. cubense Tropical Race 4 in Pakistan
Source: Front Microbiol. 2026 Oct 20;17:1957002. doi: 10.3389/fmicb.2026.1957002 (PMC13727561; doi:10.3389/fmicb.2026.1957002)
Supplement: Supplementary file 1 [file Table_1.DOCX]

| Supplementary table 1: Origin of bacterial isolates | | | | | | | | | |
| --- | --- | --- | --- | --- | --- | --- | --- | --- | --- |
| Sample No | **Name of farms** | **Coordinates** | | **Date of sampling** | **No. of plants** | **Tissue Source** | **Dilution** | **Bacteria code name** | |
|  |  | **Longitude** | **Latitude** |  |  |  |  | **Old code names** | **New code names** |
| 1 | Tando jam Matiari Road Farm 2 (TJMF 2) | 25.543912 | 68.57134 | Aug-21 | 2 | Soil | 0.3 | A3 | P2.S1 |
| 2 |  |  |  |  |  |  |  | A4 | P2.S2 |
| 3 |  |  |  |  |  |  |  | A5 | P2.S3 |
| 4 |  |  |  |  |  |  |  | A10 | P2.S4 |
| 5 |  |  |  |  |  |  |  | A16 | P2.S5 |
| 6 |  |  |  |  |  |  | 0.1 | A19 | P2.S6 |
| 7 |  |  |  |  |  | Root | 0.1 | A38 | P2.A1 |
| 8 |  |  |  |  |  |  |  | A40 | P2.A2 |
| 9 |  |  |  |  |  |  |  | A41 | P2.A3 |
| 10 |  |  |  |  |  |  | 0.3 | A41b | P2.A4 |
| 11 | PARC Farm Nigab1 Variety (PFNVB) BB1 | 33.67458 | 73.12345 | Aug-21 | 3 | Soil | 0.3 | B5 | P3.S1 |
| 12 |  |  |  |  |  |  |  | B8 | P3.S2 |
| 13 |  |  |  |  |  |  |  | B9 | P3.S3 |
| 14 |  |  |  |  |  |  |  | B10 | P3.S4 |
| 15 | Tando jam Matiari Road Farm 1 (TJMF1) | 25.49403 | 68.78454 | Aug-21 | 1 | Root | 0.3 | C28 | P1.A1 |
| 16 |  |  |  |  |  |  | 0.1 | C33a | P1.A2 |
| 17 |  |  |  |  |  | Stem |  | C83 | P1.B1 |
| 18 |  |  |  |  |  |  |  | C84 | P1.B2 |
| 19 | Tando jam Matiari Road Farm 2 (TJMF 2) | 25.543912 | 68.57134 | Aug-21 | 2 | Root |  | D2 | P2.A5 |
| 20 |  |  |  |  |  |  |  | D4 | P2.A6 |
| 21 |  |  |  |  |  | Stem | 0.3 | D4a | P2.B1 |
| 22 |  |  |  |  |  | Soil | 0.1 | D10 | P2.S7 |
| 23 | PARC Farm Nigab1 Variety (PFNVB) BB1 | 33.67458 | 73.12345 | Aug-21 | 3 | Soil | 0.1 | E4a | P3.S5 |
| 24 |  |  |  |  |  |  |  | E4b | P3.S6 |
| 25 |  |  |  |  |  |  |  | E8 | P3.S7 |
| 26 |  |  |  |  |  | Root |  | E9 | P3.A1 |
| 27 |  |  |  |  |  | Soil |  | E10 | P3.S8 |
| 28 |  |  |  |  |  |  |  | E11 | P3.S9 |
| 29 |  |  |  |  |  | Root |  | E11b | P3.A2 |
| 30 |  |  |  |  |  | Stem |  | E15 | P3.B1 |
| 31 |  |  |  |  |  | Root |  | E19 | P3.A3 |
| 32 |  |  |  |  |  | Stem |  | E21 | P3.B2 |
| 33 |  |  |  |  |  | Root |  | F1 | P3.A4 |
| 34 |  |  |  |  |  | Soil |  | F2 | P3.S10 |
| 35 |  |  |  |  |  | Soil |  | F3 | P3.S11 |
| 36 |  |  |  |  |  | Soil |  | F4 | P3.S12 |
| 37 |  |  |  |  |  | Soil |  | F5 | P3.S13 |
| 38 | PARC Farm Nigab 1 Variety BSV (PFNSVB) | 33.67458 | 73.12345 | Aug-21 | 4 | Soil | 0.1 | S6 | P4.S1 |
| 39 |  |  |  |  |  |  |  | S12 | P4.S2 |
| 40 |  |  |  |  |  |  |  | S14 | P4.S3 |
| 41 |  |  |  |  |  |  |  | S15 | P4.S4 |
| 42 |  |  |  |  |  | Root | 0.1 | S14a | P4.A1 |
| 43 |  |  |  |  |  |  |  | S19 | P4.A2 |
| 44 | Rashidi Farm | 26.708814 | 68.67754 | 18-Oct-23 | 5 | Soil | 0.1 | A1 | P5.S1 |
| 45 |  |  |  |  |  |  |  | A2 | P5.S3 |
| 46 |  |  |  |  |  |  |  | A3 | P5.S3 |
| 47 |  |  |  |  |  |  |  | A4 | P5.S4 |
| 48 |  |  |  |  |  |  |  | A5 | P5.S5 |
| 49 |  |  |  |  |  |  |  | A7 | P5.S6 |
| 50 |  |  |  |  |  |  |  | A8 | P5.S7 |
| 51 |  |  |  |  |  |  |  | A9 | P5.S8 |
| 52 |  |  |  |  |  |  |  | A10 | P5.S9 |
| 53 |  |  |  |  |  | Stem | 0.1 | B3 | P5.B1 |
| 54 |  |  |  |  |  |  |  | B5 | P5.B2 |
| 55 |  |  |  |  |  |  |  | B8 | P5.B3 |
| 56 |  |  |  |  |  |  |  | B13 | P5.B4 |
| 57 |  |  |  |  |  |  |  | B15 | P5.B5 |
| 58 |  |  |  |  |  |  |  | B17 | P5.B6 |
| 59 |  |  |  |  |  |  |  | C1 | P5.B7 |
| 60 | Hanif Farm | 25.425753 | 68.612045 | 19-Oct-23 | 6 | Soil | 0.1 | D1 | P6.S1 |
| 61 |  |  |  |  |  |  |  | D2 | P6.S2 |
| 62 |  |  |  |  |  |  |  | D3a | P6.S3 |
| 63 |  |  |  |  |  |  |  | D3b | P6.S4 |
| 64 |  |  |  |  |  |  |  | D4a | P6.S5 |
| 65 |  |  |  |  |  |  |  | D6 | P6.S6 |
| 66 |  |  |  |  |  |  |  | D7 | P6.S7 |
| 67 |  |  |  |  |  |  |  | D8 | P6.S8 |
| 68 |  |  |  |  |  |  |  | D10 | P6.S9 |
| 69 |  |  |  |  |  |  |  | D12 | P6.S10 |
| 70 |  |  |  |  |  |  |  | D14 | P6.S11 |
| 71 |  |  |  |  |  |  |  | D16 | P6.S12 |
| 72 |  |  |  |  |  |  |  | D20 | P6.S13 |
| 73 |  |  |  |  |  |  |  | D21 | P6.S14 |
| 74 |  |  |  |  |  |  |  | D22 | P6.S15 |
| 75 |  |  |  |  |  |  |  | D23 | P6.S16 |
| 76 |  |  |  |  |  |  |  | D24 | P6.S17 |
| 77 |  |  |  |  |  |  |  | D25 | P6.S18 |
| 78 |  |  |  |  |  |  |  | D26 | P6.S19 |
| 79 |  |  |  |  |  | Stem | 0.1 | E1 | P6.B1 |
| 80 |  |  |  |  |  |  |  | E2 | P6.B2 |
| 81 |  |  |  |  |  |  |  | E3 | P6.B3 |
| 82 |  |  |  |  |  |  |  | E4 | P6.B4 |
| 83 |  |  |  |  |  |  |  | E5 | P6.B5 |
| 84 |  |  |  |  |  |  |  | E6 | P6.B6 |
| 85 |  |  |  |  |  |  |  | E7 | P6.B7 |
| 86 |  |  |  |  |  |  |  | E8 | P6.B8 |
| 87 |  |  |  |  |  |  |  | E9 | P6.B9 |
| 88 |  |  |  |  |  |  |  | E10 | P6.B10 |
| 89 |  |  |  |  |  |  |  | E11 | P6.B11 |
| 90 |  |  |  |  |  |  |  | E12 | P6.B12 |
| 91 |  |  |  |  |  |  |  | E19 | P6.B13 |
| 92 |  |  |  |  |  |  |  | C2 | P6.B14 |
| 93 |  |  |  | 19-Oct-23 | 7 | Soil | 0.1 | F1 | P7.S1 |
| 94 |  |  |  |  |  |  |  | F2 | P7.S2 |
| 95 |  |  |  |  |  |  |  | F3 | P7.S3 |
| 96 |  |  |  |  |  |  |  | F4 | P7.S4 |
| 97 |  |  |  |  |  |  |  | F5 | P7.S5 |
| 98 |  |  |  |  |  |  |  | F6 | P7.S6 |
| 99 |  |  |  |  |  |  |  | F7 | P7.S7 |
| 100 |  |  |  |  |  |  |  | F8 | P7.S8 |
| 101 |  |  |  |  |  | Stem | 0.1 | H1 | P7.B1 |
| 102 |  |  |  |  |  |  |  | H2 | P7.B2 |
| 103 |  |  |  |  |  |  |  | H3 | P7.B3 |
| 104 |  |  |  |  |  |  |  | H4 | P7.B4 |
| 105 |  |  |  |  |  |  |  | H6 | P7.B5 |
| 106 |  |  |  |  |  |  |  | H7 | P7.B6 |
| 107 |  |  |  |  |  |  |  | H9 | P7.B7 |
| 108 |  |  |  |  |  |  |  | H10 | P7.B8 |
| 109 | Rashidi Farm | 26.708814 | 68.67754 | 20-Oct-23 | 8 | Soil | 0.1 | R1 | P8.S1 |
| 110 |  |  |  |  |  |  |  | R2 | P8.S2 |
| 111 |  |  |  |  |  |  |  | R3 | P8.S3 |
| 112 |  |  |  |  |  |  |  | R6 | P8.S4 |
| 113 |  |  |  |  |  |  |  | R7 | P8.S5 |
| 114 |  |  |  |  |  |  |  | R9 | P8.S6 |
| 115 |  |  |  |  |  |  |  | R11 | P8.S7 |
| 116 |  |  |  |  |  |  |  | R14 | P8.S8 |
| 117 |  |  |  |  |  |  |  | R15 | P8.S9 |
| 118 |  |  |  |  |  |  |  | R16 | P8.S10 |
| 119 |  |  |  |  |  |  |  | R17 | P8.S11 |
| 120 |  |  |  |  |  |  |  | R18 | P8.S12 |
| 121 |  |  |  |  |  |  |  | R19 | P8.S13 |
| 122 |  |  |  |  |  |  |  | R20 | P8.S14 |
| 123 |  |  |  |  |  |  |  | R21 | P8.S15 |
| 124 |  |  |  |  |  |  |  | R22 | P8.S16 |
| 125 |  |  |  |  |  |  |  | R23 | P8.S17 |
| 126 |  |  |  |  |  |  |  | R24 | P8.S18 |
| 127 |  |  |  |  |  |  |  | R25 | P8.S19 |
| 128 |  |  |  |  |  |  |  | R27 | P8.S20 |
| 129 |  |  |  |  |  |  |  | R29 | P8.S21 |
| 120 |  |  |  |  |  |  |  | R31 | P8.S22 |
| 131 |  |  |  |  |  | Stem | 0.1 | S2 | P8.B1 |
| 132 |  |  |  |  |  |  |  | S3 | P8.B2 |
| 133 |  |  |  |  |  |  |  | S4 | P8.B3 |
| 134 |  |  |  |  |  |  |  | S6 | P8.B4 |
| 135 |  |  |  |  |  |  |  | S10 | P8.B5 |
| 136 |  |  |  |  |  |  |  | S12 | P8.B6 |
| 137 |  |  |  |  |  |  |  | S13 | P8.B7 |
| 138 |  |  |  |  |  |  |  | S14 | P8.B8 |
